# Evaluating the impact of improved maize varieties on agricultural productivity and technical efficiency among smallholder farmers in the Eastern Cape, South Africa: an empirical analysis

**DOI:** 10.1080/21645698.2025.2476667

**Published:** 2025-03-19

**Authors:** Lelethu Mdoda, Nthabeleng Tamako, Lungile S. Gidi, Denver Naidoo

**Affiliations:** a Discipline of Agricultural Economics, University of KwaZulu-Natal, Scottsville, Pietermaritzburg, South Africa; b African Centre for Food Security (ACFS), University of KwaZulu-Natal, Scottsville, Pietermaritzburg, South Africa; c Department of Agricultural Economics and Animal Science, University of Limpopo (Turfloop Campus), Sovenga, South Africa

**Keywords:** Adoption rates, endogenous switching regression, improved maize varieties, productivity, smallholder farmers, stochastic meta-frontier approach, technical efficiency

## Abstract

Agriculture is essential to South Africa’s economy, and maize is a crucial crop for smallholder farmers in the Eastern Cape. Traditional maize varieties face challenges related to productivity and resilience, prompting the promotion of Improved Maize Varieties (IMVs) to enhance yields and efficiency. This study investigates the impact of IMV adoption on agricultural productivity and technical efficiency in the region, addressing a gap in empirical evidence. Using a multistage random sampling approach, data was collected from 150 smallholder maize farmers and analyzed using stochastic production frontier, endogenous switching regression models, and the stochastic meta-frontier model. The study results reveal that 62% of the farmers are male, averaging 53 years old, and manage about four hectares with a mean monthly income of ZAR 3,562.13. Challenges, such as rainfall shortages and limited access to credit, hinder IMV adoption, although high access to extension services and diverse input use positively affect productivity. The adopted IMVs by farmers, including open-pollinated, hybrid, and genetically modified (GM) varieties, significantly boost maize yields and farm returns – yielding an average increase of 1.92 metric tonnes/ha and returns of ZAR 468.01 per hectare. Key adoption factors are education, farm size, and access to seeds and extension services, whereas barriers include market distance and family size. Technical efficiency is generally high at 74%, with farm size, seed, pesticides, agrochemicals, and fertilizers positively impacting maize production, whereas family labor negatively affects it. Factors such as age, education, and access to services significantly reduce technical inefficiency, while herd size, off-farm income, and distance to the market have mixed effects. The stochastic meta-frontier approach reveals that smallholder farmers adopting improved technologies show higher mean technical efficiency, indicating that advanced methods contribute to better resource use and productivity than traditional systems. This study suggests that targeted support is needed for farmers, enhancing access to extension services, affordable seeds, financial support, and investing in infrastructure and education can further improve adoption rates, technical efficiency, and overall productivity. Promoting improved technologies such as maize varieties will enhance the technical efficiency of farms, regardless of their adoption status. It would be key to improving overall agricultural productivity and farm household incomes.

## Introduction

Agricultural productivity is a crucial driver of economic growth and food security, especially in developing regions where a substantial portion of the population relies on agriculture for their livelihoods. In this context, enhancing agricultural productivity improves food availability and serves as a vital pathway to poverty alleviation and rural development. Nevertheless, smallholder farmers often face significant challenges, including limited resource access, outdated farming practices, and climate change impacts. These issues hinder the potential for increased productivity and technical efficiency in agricultural practices.

One promising strategy to overcome these challenges is the adoption of Improved Maize Varieties (IMVs), which have been promoted for their ability to significantly boost crop yields and improve the technical efficiency of smallholder farmers. Despite extensive research on adopting IMVs, a gap remains in understanding their specific effects on agricultural productivity and technical efficiency in distinct regional contexts.

This study identifies the following key research issues:

1. The extent to which IMV adoption influences agricultural productivity and technical efficiency among smallholder farmers in the Eastern Cape Province.
2. The socio-economic factors that affect the adoption rate of IMVs and the subsequent impact on farmer performance.

The expected contributions of this study are multifaceted. Firstly, it will provide empirical evidence that highlights the effectiveness of IMVs in enhancing agricultural performance, thereby filling a gap in the current literature. Policymakers and agricultural development organizations are the primary beneficiaries of this research, as the findings will offer critical insights that can inform policy decisions and the design of targeted agricultural development programs. Furthermore, smallholder farmers will benefit from a better understanding of how adopting improved varieties can lead to improved yields and efficiency, ultimately contributing to improved livelihoods and food security.

## Background

Maize is a staple crop in many parts of the world, particularly in sub-Saharan Africa (SSA), where it plays a crucial role as a primary source of food and income for millions of smallholder farmers. Maize is a versatile and widely cultivated cereal crop that has become a staple food in many regions, especially sub-Saharan Africa. Maize is the most important staple crop in terms of the harvested area and production in the region. The maize crop is mainly used for consumption and feeding to sustain rural livelihoods.^1^ It is a source of calories for millions and a critical component of the agricultural economy, providing income and employment opportunities for smallholder farmers. Despite their importance, smallholder farmers in sub-Saharan Africa face numerous challenges in maize production, especially in these climate and environmental conditions.

Maize is a staple crop in many parts of the world, particularly in sub-Saharan Africa (SSA), where it plays a crucial role as a primary source of food and income for millions of smallholder farmers. In South Africa, maize emerges as the most widely cultivated field crop. It serves as a central pillar for food security and the livelihoods of a significant portion of the population. Approximately 60% of South Africa’s maize is white maize for human consumption, while the remaining 40% is yellow maize, primarily used for animal feed.^2^ The country produces between 15 million and 17 million tons of maize annually, with a cultivated area ranging from 2.5 million to 3 million hectares.^3^ However, maize production is highly concentrated in specific provinces, with the Free State, North West, and Mpumalanga accounting for the majority. At the same time, the Eastern Cape contributes only 1% of the national production.^2^

The selection of maize as the core focus of this study is justified by its significant socio-economic impact in South Africa. Furthermore, understanding the role of Improved Maize Varieties (IMVs), specifically in the Eastern Cape, provides insights that may apply to similar regions facing agricultural challenges. Although the findings from this study may be context-specific, they may also hold generalizable implications for other drought-prone areas in southern Africa where similar socio-economic factors affect maize production.

Climate change has increasingly threatened agricultural productivity across SSA, mainly maize. South Africa, characterized by semi-arid conditions, faces rising temperatures and unpredictable rainfall patterns, further complicating the agricultural landscape. Studies suggest that the impacts of climate change could lead to significant reductions in maize yields, with projections estimating declines of up to 24% in the next decade, particularly exacerbating food insecurity in vulnerable regions.^4,5^ Adopting climate-resilient agricultural practices, mainly through IMVs, becomes crucial in mitigating these challenges.

IMVs are designed to be more resilient to biotic and abiotic stresses and are positioned to offer higher yield potential compared to traditional varieties. Research shows that introducing such improved varieties can significantly enhance total maize production and provide an opportunity to develop better household welfare outcomes. Across various studies in SSA, households that adopt IMVs tend to experience higher food security and increased income levels.^6,7^ IMVs have been linked to improved technical efficiency, defined as producing greater output from a given set of inputs, thus fostering a sustainable agricultural system.

The historical context of maize production in South Africa has highlighted the issue of low productivity levels attributed to ineffective agricultural practices, inadequate access to improved seeds, and adverse climatic conditions.^8,9^ Despite the promotion of IMVs, the disparity in adoption rates across different regions remains noticeable due to environmental factors, socio-economic barriers, and varying levels of technical knowledge among farmers. Additionally, smallholder farmers’ multifaceted challenges, including limited access to credit and extension services, further complicate the effective utilization of IMVs.

Recognizing that the Eastern Cape exhibits unique socio-economic, environmental, and agricultural characteristics that influence IMV adoption and effectiveness is essential. Smaller-scale farming operations and significant reliance on subsistence farming mark this province’s agricultural landscape. Understanding how these factors impact the adoption and efficacy of IMVs will provide valuable lessons for improving agricultural practices in the Eastern Cape and potentially in other similarly challenged regions.

Through a comprehensive examination of IMVs’ impact on agricultural productivity and technical efficiency, this study aims to inform stakeholders about the potential effectiveness of adopting these technologies. Greater awareness can lead to more informed policy decisions and agricultural development programs that directly benefit smallholder farmers, ultimately contributing to improved food security and rural economic growth in South Africa.

## Theoretical Framework

The uptake of agricultural technologies hinges on maximizing the anticipated utility or advantages for the farmer. This process frequently requires careful equilibrium across various economic, environmental, and risk factors. Smallholder farmers simultaneously make direct production and consumption decisions, a necessity driven by market imperfections, where they must balance profit and utility maximization. The study on the productivity impact of adopting improved maize varieties (IMVs) and technical efficiency in the Eastern Cape Province, South Africa, employed the Random Utility Framework (RUF) to examine this complex decision-making process. This approach was chosen because smallholder farmers’ decisions to adopt IMVs are deeply intertwined with their perceptions and comprehension of IMVs, transforming farm decisions into a choice problem. The RUF, extensively utilized in studies focusing on farmers’ decisions to adopt innovative techniques, has been employed in similar research endeavors.^10–13^ In economic analysis, RUF is a potent tool for researchers to investigate smallholder farmers’ decision-making densities. In the agricultural domain, RUF plays a pivotal role, especially in farmers’ adoption of IMVs and technical efficiency.

The RUF is a robust framework for understanding the decision-making process behind farmers’ adoption of IMVs. In this model, farmers are considered rational actors who weigh the potential benefits and costs of adopting IMVs to maximize utility. Several factors influence the utility of farmers’ IMV adoption. Expected yield gains are crucial, as higher yields translate to increased profitability and improved farmer livelihoods. Additionally, reducing the production risks associated with IMVs, such as enhanced resistance to pests and diseases or better adaptability to environmental conditions, contributes to farmers’ utility. Moreover, subjective preferences, including attitudes toward new technologies and cultural considerations, also shape farmers’ perceptions of the utility of IMVs adoption. RUF enables researchers to untangle the intricacies of farmers’ decisions regarding IMV adoption and resource allocation to enhance productivity. Through this framework, economists can assess the trade-offs inherent in IMV adoption by^,^ weighing potential yield increases against the associated costs and risks.^13,14^ Moreover, the random utility framework facilitates an understanding of how farmers optimize resource allocation, considering factors such as land, labor, capital, and technology, ultimately aiming to maximize agricultural output. Furthermore, the random utility framework offers insights into the socio-economic dynamics shaping farmers’ decision-making processes. It allows researchers to explore heterogeneity among farmers in terms of preferences, risk attitudes, access to information, and institutional constraints. This nuanced understanding is essential for designing effective agricultural policies and interventions to promote IMV adoption and improve technical efficiency. Moreover, by identifying the drivers of farmers’ decisions, policymakers can tailor their strategies to address the barriers or incentives hindering the widespread adoption of IMVs. Applying the random utility framework to study the productivity impact of IMVs adoption and technical efficiency provides valuable insights into the complex interplay between factors influencing agricultural decision-making. This offers guidance for fostering sustainable agricultural development.

This study assumes that smallholder farmers profoundly understand their primary agricultural challenges and articulate their decisions and choices regarding various IMVs. This assumption implies that farmers’ preferences are shaped by their inherent expectations of the costs and benefits of alternative interventions, considering their available resources. The expectation is that farmers will logically express their preferences in a manner that enhances both productivity and overall well-being. These decisions can be conceptualized through a utility function, allowing the study to frame the decision-making process as one aimed at maximizing utility. Based on the premise that farmers’ available information consists solely of ranking choice scenarios regarding IMV adoption, this study derives a principle for evaluating individual farmers’ productivity and livelihoods.^15^ Observing farmers’ choices among various IMV interventions can give valuable insights into the utility ranking of alternatives. However, when farmers are tasked with expressing their preferences for IMVs, natural ordering among the alternatives may be absent, and no assumption can be made regarding the monotonic relationship between an underlying latent variable and the observed outcomes for ordering interventions. In such instances, a common framework employed to structure diverse probabilities is the random utility framework, where the utility of each alternative comprises a linear function of observable individual characteristics plus an additive error term.^16^ By imposing suitable distributional assumptions on the error terms, this approach provides manageable expressions for the probabilities a model implies. Following the stated choice method, the econometric model used to investigate the determinants of farmers’ adoption of IMVs in this study was a random utility framework.

By applying RUF to study the adoption of IMVs, farmers can identify the determinants driving adoption decisions and quantify the relative importance of different factors. This enables policymakers and agricultural extension services to tailor interventions effectively, address barriers to adoption, and promote the uptake of IMVs among farmers. Ultimately, a deeper understanding of the decision-making process through the RUM framework can lead to more targeted and impactful agricultural development strategies, fostering sustainable productivity growth and improving food security in maize-dependent regions.

Consider a scenario in which a farmer benefits from adopting the available IMVs participating resources. The IMVs on the farm can be denoted by j, where j = 1, signifying whether the farmer is willing to adopt the IMVs, and j = 0 otherwise. The farm household’s resource endowment is represented by w, whereas vector x encapsulates other observable attributes that may influence the desirability of the adoption of IMVs.

Suppose the farmer chooses the IMVs as their choice in the farm. In that case, their utility is expressed as $U_{1}=U\left(1,w{\;}^{\prime },x\right)$, whereas if the farmer does not select IMVs in the farm, $U_{0}=U\left(0,w{\;}^{\prime },x\right)$. According to economic theory, farmers are expected to select IMVs that maximize their utility, considering their constraints. In line with the typical specification of utility functions, this study assumes an additively separable utility function comprising deterministic and stochastic components, in which the deterministic component is presumed to exhibit linearity in the explanatory variables.

(1) $$U_{1}=U\left(1,w{\;}^{\prime },x\right)=V\left(1,w{\;}^{\prime },x\right)+\varepsilon_{1}$$

(2) $$U_{0}=U\left(0,w{\;}^{\prime },x\right)=V\left(0,w{\;}^{\prime },x\right)+\varepsilon_{0}$$

Where

$U_{j}$(.) is the utility from the IMV, $V_{j}$(.) is the deterministic part of the utility, and $\varepsilon_{j}$ is the stochastic component representing the component of utility known to the farmers but unobservable to the economic investigator. Farmers are assumed to know their resource endowment, w, and the implicit cost of participating in the program in terms of engagement of their resources and can decide whether to adopt IMVs. Let the farmer’s implicit cost of adoption be represented by A. Therefore, the farmer will prefer a development program if

(3) $$U_{1}\left(.\right)\geq U_{0}\left(.\right),V\left(1,w-A;x\right)+\varepsilon_{1}\geq V\left(0,w;x\right)+\varepsilon_{0}$$

The presence of the random component permits probabilistic statements about the decision-makers’ behavior. If the farmer chooses the IMVs, the probability distribution is given by:

(4) $$P_{1}=\mathrm{Pr}\left(\mathrm{choose}\right)=\mathrm{Pr}\left(V\left(1,w-A;x\right)+\varepsilon_{1}\geq V\left(0,w;x\right)+\varepsilon_{0}\right)$$

and if the farmer did not choose the IMVs,

(5) $$P_{0}=\mathrm{Pr}\left(\mathrm{notchosen}\right)=\mathrm{Pr}\left(V\left(0,w;x\right)+\varepsilon_{0}\right)\geq \mathrm{Pr}V\left(1,w-A;x\right)+\varepsilon_{1}$$

We hypothesize that adopting Improved Maize Varieties (IMVs) holds significant promise for augmenting maize productivity and enhancing net farm income. We assume that the rise in maize productivity and net farm income can be represented as a linear function of IMVs. With the assumption that the deterministic component of the utility function is linear in the explanatory variables, the utility functions in (1) and (2) can be expressed as $U_{1}=\beta_{1}^{\prime }X_{i}+\varepsilon_{1}$, and $U_{0}=\beta_{0}^{\prime }X_{i}+\varepsilon_{0}$ respectively, and the probabilities in Equation 4 and 5 can be given as:

(6) $$\mathrm{Pr}\left(\mathrm{choose}\right)=\mathrm{Pr}\left(U_{1}\left(.\right)\geq U_{0}\left(.\right)\right)=\mathrm{Pr}(\beta_{1}^{\prime }X_{i}+\varepsilon_{1}\geq \beta_{0}^{\prime }X_{i}+\varepsilon_{0}=\mathrm{Pr}(\beta_{1}^{\prime }X_{i}-\beta_{0}^{\prime }X_{i}\geq \varepsilon_{0}-\varepsilon_{1}$$

## Methodology

### Description of the Study Area

**Figure 1.**
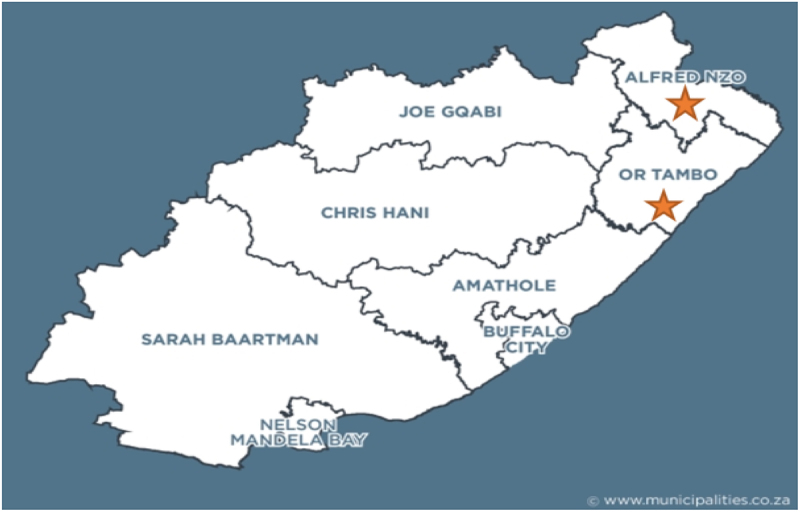
Map showing the study areas within the Eastern Cape Province, South Africa. The orange stars indicate the or Tambo and Alfred Nzo district municipalities.

This study was conducted in the Eastern Cape Province of South Africa, focusing on OR Tambo and Alfred Nzo District Municipalities. Figure 1 above shows the study area. Both municipalities are Category C, located in the eastern and northeastern corners of the Eastern Cape. The Eastern Cape is often referred to as a world in one province. Eastern Cape Province is the second largest province in South Africa after Northern Cape Province. It offers everything from snow skiing to sunny beaches, game research, and fascinating history. Located in the easternmost part of South Africa, an area of almost 170,000 square kilometers of diverse landscape. The province has a population of 6,562,053, and most people reside in rural areas and derive their livelihoods from practicing agriculture.^17^ The persistent poverty in this province lies below the poverty line.^18,19^ The province offers an agricultural background (crop, vegetable, citrus, and livestock farming) spread with a trivial number of agro-industrial and eco-tourism infrastructures. Agriculture is their primary activity for livelihood, given the high poverty rates and food insecurity, which hampers the province. The Eastern Cape is unique among South Africa’s nine provinces, encompassing all seven biomes or ecological zones within its borders. This diverse range of ecosystems provides the province with various climates, enabling a broad spectrum of agricultural and economic activities. Additionally, the Eastern Cape enjoys more days of sunshine than any other province in South Africa, further enhancing its agricultural and industrial potential. The province is active in maize production, and smallholder farmers dominate it.

The Eastern Cape, primarily a rural region, relies heavily on smallholder agriculture, with maize cultivation playing a pivotal role in food security and income generation for many households. The province’s temperate climate, with its distinct wet and dry seasons, provides an environment conducive to agriculture, particularly during the rainy season when maize thrives. However, this dependency on seasonal rainfall makes the region, especially the OR Tambo and Alfred Nzo Districts, highly vulnerable to the impacts of climate variability (Mdoda, 2020). Periodic droughts, which have become more frequent and severe due to changing climate patterns, threaten agricultural productivity. These droughts reduce water availability for irrigation, hinder crop growth, and diminish yields, exacerbating food insecurity and economic hardship for many smallholder farmers under resource constraints.^20^ As a result, the region faces a complex challenge of balancing the benefits of its agricultural potential with the risks posed by unpredictable weather patterns, which calls for the implementation of climate-resilient agricultural practices, improved water management strategies, and enhanced infrastructure to support sustainable farming in the face of such climate-related challenges.

The OR Tambo and Alfred Nzo Districts are known for their high concentration of smallholder maize farmers. Although agriculture is a crucial economic sector, contributing to 12% and 3.5% of the respective districts’ economies, the growth potential is hampered by reduced risk, better economic outcomes, socioeconomic factors (knowledge and education, market demand, social networks, tenure security, and governmental support), climatic challenges, long term sustainability, and pest diseases resistance, which are the main underlining reasons for the importance of adopting IMVs for enhanced productivity, food security, profitability and reduced poverty.^20,21^ The cultural context in the OR Tambo and Alfred Nzo districts plays a significant role in shaping the adoption of improved maize varieties (IMVs). Farmers in these regions are more likely to embrace new agricultural technologies if they perceive these innovations as compatible with their traditional farming practices and cultural values. This cultural alignment fosters greater acceptance and trust in improved maize varieties, making farmers more willing to incorporate them into their existing systems. Furthermore, institutional factors, such as the availability of local seed distributors, extension services, and farmer support networks, are essential in facilitating the adoption process. Access to reliable information, technical assistance, and affordable inputs enhances farmers’ ability to obtain and effectively use improved maize varieties. Therefore, the interaction between cultural norms and institutional support structures plays a crucial role in determining the success and scalability of agricultural innovations in these districts. Agricultural activities in the area are influenced by limited access to water resources, poor soil quality in some areas, and challenges related to access to markets and credit facilities.^20^ On the other hand, Alfred Nzo District, which lies adjacent to OR Tambo, shares similar socio-economic characteristics but is more rural with lower population density and greater reliance on agricultural extension services for knowledge dissemination on IMVs.^21^

The study targeted OR Tambo and Alfred Ndzo districts in the Eastern Cape province due to their high involvement in maize production and a high rate of genetically modified maize adoption. The areas with high adoption rates of improved maize varieties (IMVs) were specifically targeted for data collection because they provide a valuable opportunity to understand the key factors driving successful adoption and identify best practices that can be replicated in other provinces. These areas serve as exemplary models where the adoption of IMVs has yielded positive outcomes, offering insights into the socio-economic, cultural, and institutional factors that facilitate widespread use. Focusing on these districts with higher adoption rates, the study captured a richer and more relevant data set highlighting the conditions under which farmers are more likely to adopt new technologies. Additionally, targeting high-adoption areas like these two districts allows for a more precise analysis of the impact of IMVs on agricultural productivity, technical efficiency, and overall livelihoods. In these districts, adoption success is often attributed to the effective interaction of various elements, such as access to credit, adequate training, market infrastructure, and supportive government policies. Studying these areas helps identify which interventions or factors have had the greatest influence on adoption, providing a clear framework for scaling up successful strategies to other, less-adopting areas. Hence, OR Tambo and Alfred Nzo district municipalities were chosen as the study sites in the Eastern Cape province. A cross-sectional research design was used to gather the required data for this study.

### Sampling Procedure and Sample Size

This study utilized a combination of purposive sampling and multi-stage random sampling procedures to ensure a comprehensive and representative analysis. Initially, purposive sampling was employed to select the study area and two specific districts: the OR Tambo and Alfred Nzo District Municipalities within the Eastern Cape Province of South Africa. This choice was informed by extensive literature highlighting the Eastern Cape Province as a prominent region in South Africa for adopting Improved Maize Varieties (IMVs) to enhance maize productivity. Furthermore, these two districts were chosen because of the high concentration of smallholder maize farmers who have adopted IMVs, making them ideal sites for investigating the effectiveness and impact of these varieties. Following the purposive selection of the districts, multi-stage random sampling was applied to refine the study’s focus. The sampling process involved the following stages:

1. **District Selection**: The initial stage involved purposively selecting the two districts based on their maize production potential and the prevalence of farmers utilizing improved technologies, particularly IMVs. Despite the high adoption rates of IMVs, detailed knowledge regarding their technical efficiency and impact on maize yields in these districts is lacking.
2. **Ward and Community Selection**: The second stage focused on selecting specific wards and communities within the chosen districts. This step aimed to identify the areas where the selected smallholder farmers were located, ensuring that the sample was representative of the farming population in these regions.
3. **Farmer Selection**: The final stage involved randomly selecting smallholder maize farmers from the identified wards and communities. This random selection process was designed to achieve a final sample size of 150 farmers, as determined using Cochran’s formula. The sample size of 150 respondents was based on statistical considerations, such as ensuring a confidence level of 95% and an acceptable margin of error (usually around 5%). The calculated sample size reflected the diversity of the larger population in terms of key variables such as farm size, gender, age, experience, access to resources, and adoption rates of improved maize varieties (IMVs). The main goal of having 150 was to ensure that the sample captures a broad spectrum of experiences and characteristics within the study area, especially the adopters and non-adopters of IMVs. The calculated sample size provided a solid and statistically sound representation of the study area’s total population of maize farmers.

The sample size was calculated to ensure statistical validity and reliability despite the absence of precise data on the total number of farmers within the study sites who had adopted IMVs. These sampling procedures were instrumental in obtaining a representative sample. They provided a robust basis for analyzing improved maize varieties’ technical efficiency and impact on maize yields in the selected districts.

### Data Collection

The study used primary data gathered through face-to-face interviews using a semi-structured questionnaire. The questionnaire was translated into a local dialect with trained enumerators to ensure that participants could easily comprehend the questions. Before data collection, a pre-testing phase was conducted to train the enumerators, assess the reliability of the questionnaire, evaluate the time required for responses, and ensure that the questionnaire addressed all the necessary aspects. The data collection process focused on several key areas: demographic and institutional characteristics of maize farmers, types of improved maize varieties (IMVs) adopted by farmers, input data (including farm size, land area, labor, fertilizer use, and yields), and harvest output quantities. Additionally, information was gathered on the sources of IMVs, the benefits of using IMVs compared to traditional seeds, and the challenges farmers face when adopting and using IMVs. Data were collected from adopters and non-adopters within the study area to provide a comprehensive overview of the impact and adoption of IMVs.

### Data Analysis

The study employed a structured questionnaire to gather data, which were then systematically coded in an Excel spreadsheet. These data were subsequently imported into STATA 17 and SPSS version 25 for comprehensive analysis. Descriptive statistics were utilized to evaluate demographic characteristics, adoption of IMVs, the benefits associated with IMV adoption, and the challenges encountered. This analysis uses means, frequencies, percentages, tables, and pie charts to provide a clear and detailed overview. A stochastic production frontier model is employed to assess the productivity efficiency of farmers using IMVs. This model allowed for the estimation of the technical efficiency of maize production by adopting improved varieties. Additionally, an endogenous switching regression model is applied to evaluate the impact of IMV adoption on farmers’ welfare. This approach facilitated a nuanced understanding of how IMV adoption affects farmers’ well-being and economic outcomes. Combining these analytical techniques thoroughly examines the effectiveness and implications of adopting improved maize varieties in the study area.

### Stochastic Production Frontier (SPF)

Smallholder maize farmers strive to optimize production, minimize expenses, and maximize profits. While each producer endeavors to achieve these goals, not all may succeed equally. Some farmers demonstrate greater operational efficiency despite utilizing similar resources and adopting technology. Through econometric estimation techniques, it is possible to distinguish whether deviations from optimal choices stem from inefficiency or random external factors. Such techniques facilitate the calculation of efficiency estimates or scores for individual producers, enabling the identification of those requiring interventions and corrective actions. This study employs the approach of estimating a stochastic frontier production function, as outlined by previous studies that used this model.^18,22,23^

This study relies on the stochastic production frontier (SPF) methodology to evaluate technical efficiency and how it is influenced by IMV adoption. SPF) has found widespread application in the agricultural sector for evaluating technical efficiency (TE).^24^ It has been favored in numerous empirical investigations because of its capacity to handle data variability and inefficiencies. The stochastic production frontier method involves making a predetermined assumption about the functional form of input-output relationships and the distributional characteristics of inefficiency terms.^25,26^ The stochastic production frontier is a powerful econometric model that analyzes production processes, particularly for agricultural and industrial production.^27,28^ This is a sophisticated extension of the deterministic production frontier, assuming that observable factors explain all production inefficiencies. The stochastic production frontier acknowledges that unobservable factors or random shocks affect production efficiency. This could be due to managerial skills, technological advancements, weather conditions, or other external influences. The model incorporates these random elements into its framework, allowing a more realistic representation of the production process.

Estimating the stochastic production frontier involves two main steps: estimating the production function parameters through standard regression techniques to understand the input-output relationship without inefficiencies.^29^ Secondly, the inefficiency effects can be estimated using statistical methods such as maximum likelihood estimation (MLE) or Bayesian techniques to capture unobserved and stochastic elements impacting production efficiency. This model offers advantages such as accurately representing production inefficiencies by incorporating stochastic elements, identifying sources of inefficiency through decomposition into random and technical (components), and exhibiting robustness to various statistical assumptions and error term distributions, catering to diverse applications.

The Stochastic Production Frontier (SPF) utilizes the Cobb-Douglas production function as a fundamental component in modeling production processes. The Cobb-Douglas production function is a widely used mathematical representation of the relationship between inputs and outputs in production, particularly in economics. The Cobb-Douglas production function serves as the deterministic component of the model, representing the underlying production technology and the relationship between inputs and outputs in the absence of inefficiencies. The SPF then extends this framework to account for inefficiencies and stochastic elements in the production process. The stochastic production frontier model combines the deterministic Cobb-Douglas production function with random error terms to capture systematic and random production efficiency variations. This allows the model to analyze the overall efficiency level sources of inefficiency, and their impact on production. The Stochastic Frontier Analysis (SFA) model is set as follows:

(7) $$y_{i}=f\left(X_{i},\beta \right)\exp\left(v_{i}-u_{i}\right)$$

where

$y_{i}$is the maize output of farmer i; $X_{i}$ is a vector of input quantities; β is a vector of parameters; $v_{i}$ is the random error term, which follows the distribution of N (0, $\sigma_{v}^{2}$); and $u_{i}$ is the term for technical inefficiency, which is assumed to follow a truncated normal distribution.

## The Technical Efficiency

It is important to highlight that input variable $X_{i}$ encompasses two crucial components: irrigation costs and the use of chemical fertilizers. The majority of the literature has detailed a non-linear correlation between these inputs and agricultural production. To account for this, quadratic terms of irrigation costs and chemical fertilizer usage were integrated into the analysis. Maize productivity was assessed through technical efficiency, which was computed using Stochastic Frontier Analysis (SPF). Technical efficiency is defined as the ratio of actual output to potential output. Within the model, technical efficiency was estimated as follows:

(8) $$TE_{i}=\frac{E\left(y_{i}l-u_{i},X_{i}\right)}{E\left(y_{i}^{*}lu_{i}=0,X_{i}\right)}=\exp\left(-u_{i}\right)TE_{i}\varepsilon \left(0,1\right)$$

where

$TE_{i}$is technical inefficiency of the ith maize growers and ε is the maize output of farmer i. For $y_{i}$, $X_{i}$,$v_{i}$, and$u_{i}$. $y_{i}$is the observed output, $y_{i}^{*}$is the frontier output, and technical efficiency assumes a value between zero (0) and one (1), (0 ≤ $TE_{i}$ ≤1). If $u_{i}$ = 0, it implies $y_{i}$= $y_{i}^{*}$, indicating that the farmers are technically efficient (100% efficient).

The parameters of the stochastic frontier function were determined using the maximum likelihood method. The estimation process of the stochastic frontier is enhanced by adopting the reparameterization method.^30^

(9) $$\sigma^{2}=\sigma_{v}^{2}+\sigma_{u}^{2}\gamma =\frac{\sigma_{u}^{2}}{\sigma^{2}}\left(0\leq \gamma \leq 1\right)$$

Predicting individual technical efficiencies involves unobservable technical inefficiency effects $u_{i}$. The best predicator of $u_{i}$is the conditional expectation of µi, given the value of

(10) $$\varepsilon_{i}=v_{i}-u_{i}$$

Gamma (γ) = $\frac{\sigma_{u}^{2}}{\sigma^{2}}$ specifies the error associated with technical inefficiency estimates. It ranges from zero (0) to one (1). Where γ = 1, deviations from the frontier are attributed to technical inefficiency. In addition, γ = 0 implies that the noise effect causes deviations whereas 0≤γ≤1 indicates that both stochastic and non-stochastic errors are present in the data.

## Endogenous Switching Regression Model

This study utilized an endogenous switching regression model to evaluate the impact of adopted improved maize varieties (IMVs) on smallholder farmers in South Africa’s Eastern Cape Province.^31,32^ This statistical approach addresses endogeneity in regression analysis, in which independent variables may be correlated with the error term, potentially leading to biased parameter estimates. While simpler methods, such as comparing means or employing ordinary least squares (OLS) regression, treat adaptation as exogenous, research suggests endogeneity, resulting in biased estimates.^33,34^ The endogenous switching regression model mitigates this issue by dynamically switching between regression models based on variable exogeneity and integrating instrumental variables to alleviate the correlation with the error term.

The endogenous switching regression model offers advantages in flexible model specification, addressing direct endogeneity and statistical efficiency through valid instrumental variables. Compared with propensity score matching, it provides more precise parameter estimates and dynamically adjusts for data complexities.^31,32^ This study favored endogeneity switching regression because of its ability to adapt to model specifications, directly tackle endogeneity, achieve statistical efficiency, and overcome limitations associated with propensity score matching. While propensity score matching is more straightforward, endogeneity switching regression offers a more robust approach, addressing endogeneity directly and leading to more efficient parameter estimates, thus making it suitable for the analytical framework of this study.

Endogeneity can arise from factors such as omitted variable bias, reverse causality, and measurement error when estimating the effects of adopted IMVs on agricultural productivity and farm returns. The endogenous switching regression model addresses this issue by switching between different regression models based on the exogeneity or endogeneity of specific variables. This method enhances the reliability of estimating the causal relationship between adopted IMVs and agricultural outcomes. The endogenous switching regression model was structured in two stages. First, smallholder crop farmers’ adoption of IMVs was addressed using the logit selection model, considering interactions between factors. Second, the impact of each outcome equation (yield and farm returns, measured as Kilograms per hectare and ZAR per hectare) regarding adopted IMVs is estimated using least-squares regressions with selectivity correction terms. This approach facilitates a comprehensive analysis that accounts for endogeneity while evaluating the effects of adaptation strategies on agricultural performance.

### Selection of Adopted IMV

The initial model involves the selection process for the adopted IMVs, represented by a binary variable. The study adopted a logit regression model to estimate the factors influencing smallholder farmers’ adoption of improved maize varieties (IMV) in the study area. The logit technique models binary outcome variables, where the response variable can take only two possible values (usually coded as 0 and 1). This model is commonly used in various fields, such as economics, sociology, political science, and epidemiology. The parameter estimates of the model are both asymptotically consistent and efficient. Logit regression lies in its ability to analyze dichotomous decisions, such as whether a farmer adopts a particular improved maize variety to enhance productivity.^35,36^ Logit regression enables the determination of choice probabilities for various categories, facilitating a deeper understanding of the decision-making processes. Additionally, the standardized coefficients align with the beta-coefficients found in ordinary least squares regression models.

Logit regression offers coefficients that can be directly interpreted as odds ratios, facilitating easier interpretation and explanation than Probit coefficients. Logit regression tends to be computationally more efficient because of the simplicity of its cumulative distribution function, leading to faster convergence in estimation algorithms.^37^ Furthermore, logit regression exhibits greater robustness to violations of normality assumptions for error terms, making it better suited for accommodating outliers and extreme observations. Lastly, logit regression enjoys broader adoption and support in software packages and statistical literature, providing researchers with more analytical resources and tools than Probit regression. Consider A* as our binary outcome variable representing the adoption of IMVs. Specifically, this model focuses on the latent variable A*, which encapsulates the anticipated advantages of opting for adaptation compared to not adapting.^31,35,38–41^ This latent variable is formulated as follows:

(11) $$A_{i}^{*}=Z_{\mathrm{i\alpha}}+{\;}_{i}\mathrm{with}A^{*}\left\{\begin{array}{c} 1\mathrm{if}A_{i}>0\\ 0\mathrm{otherwise} \end{array}\right.$$

That is farm household i will choose to adopt IMV ($A_{i}^{*}=1$) some adaptation strategies in response to long-term changes in mean temperature and rainfall if A* > 0, and 0 otherwise. Z represents an n ^x^ m matrix of explanatory variables, α is an m × 1 vector of model parameters to be estimated, and ŋ is an n × 1 vector normally distributed with zero mean and random error terms.

## Estimation of Productivity After Adopting IMVs

The stochastic production frontier was used to measure maize productivity after adopting IMVs. The stochastic production frontier (SPF) used to estimate technical efficiency is specified as:

(12) $$y_{i}=f\left(X_{i}\beta \right)\exp\left(V_{i}-U_{i}\right)$$

where

$y_{i}$ denotes the maize output, $U_{i}$ defines a non-negative error term representing technical inefficiency and $V_{i}$indicates the effects of purely random factors on production. Technical efficiency was derived as the ratio of the observed output $y_{i}$ to the frontier output $y_{i}^{*}$ as in Eq 12:

(13) $$TE_{i}=\frac{y_{i}}{y_{i}^{*}}=\frac{f\left(X_{i}\beta \right)\exp\left(V_{i}-U_{i}\right)}{f\left(X_{i}\beta \right)\exp\left(V_{i}\right)}=\exp-U_{i};\mathrm{where}0\leq \mathrm{TE}\leq 1$$

The empirical Cobb-Douglas model specified after performing functional form test was:

(14) $$\ln y_{i}=\beta_{0}+\overset{6}{\underset{j=1}{\sum }}\beta_{j}\ln X_{\mathrm{ji}}+V_{i}-U_{i}$$

where

$X_{\mathrm{ji}}$is a vector of factor inputs including farm size (ha), labor (man-days), seed(kg), fertilizer (kg), pesticide (liters) and capital?

### Impact Assessment of Adopted IMVs on Maize Yield

This study utilizes the Empirical Treatment Regime Model (ETRM) in its empirical analysis. ETRM offers several advantages: it not only addresses selection bias stemming from the nonrandom assignment of treatment (adopters versus non-adopters) but also enables the simultaneous estimation of factors affecting the adoption of Improved Maize Varieties (IMVs) and those influencing Technical Efficiency (TE) or productivity. The third stage involves the outcome equation, where crop yield, measured in kilograms per hectare, divides the endogenous model into two distinct components.^42^ This entails implementing separate commands or production functions to analyze the decision-making process regarding small-scale crop farmers’ adoption versus non-adoption of IMVs. Moreover, it facilitates an assessment of the impact of IMV adoption on maize productivity. To assess the influence of adopting IMVs on crop yields, this study presumes that the arrays representing these outcome variables adhere to a linear relationship with explanatory factors. In this model specification, the outcome and treatment equations are defined as follows:

(15) $$TE_{i}=X_{\mathrm{i\alpha}}+y_{i}\varphi +\varepsilon_{i}$$

where

$Y_{i}$ is the vector of outcome variables (denote the predicted technical efficiency scores after estimating the stochastic frontier model in Equation (3) or the productivity of the maize farmers), and $X_{i}$ is the vector of explanatory variables such as age, education, family size, farm characteristics (e.g. farm size, location of the farm, farm machinery), soil types, and institutional and financial variables (e.g. access to extension services, climate information, and credit), while $y_{i}$ is a dummy variable capturing the adoption of IMV, α, and φ are parameters to be estimated, and εi represents the error term.

### Estimation and Identification

Given the research study’s reliance on survey data and the nonrandom nature of selection in adopted IMVs, it becomes imperative to utilize an approach that addresses selection bias effectively. Hence, this study opted for an Endogenous Switching Regression (ESR) model to mitigate the selection bias stemming from both observable and unobservable heterogeneity within the sample, drawing upon the works of previous studies.^43,44^ This model operates in two stages. First, the decision-making process regarding adoption is examined as outlined in the selection equation [Eq (1 and 2)]; secondly, two distinct equations are formulated to represent outcomes for adopters and non-adopters.

(16) $$\mathrm{Command}1\mathrm{toAdoptIMV}y_{1i}=X_{1i}\beta +\varepsilon_{1i}\mathrm{if}A_{i}=1$$

(17) $$\mathrm{Command}2\mathrm{toNottoadapt}y_{2i}=X_{2i}\beta +\varepsilon_{2i}\mathrm{if}A_{i}=0$$

where

$y_{1i}$ and $y_{2i}$, respectively represent crop yield for adopters of IMV and non-adopters, respectively, measured as kg/hectare. $X_{i}$ is the list of explanatory variables that consists of inputs, climate variables, and socioeconomic, institutional, and farm characteristics, and $\varepsilon_{1i}$ and $\varepsilon_{2i}$ are the error terms for adapters and non-adapters, respectively.

In the context of this switching regression model, selection bias arises in the error terms ε and ŋ. Assuming that the explanatory factors do not account for unobserved variables, a correlation exists between the error terms of the production and selection equations, denoted as corr (ε, ŋ) ≠ 0. The error terms ŋi, $\varepsilon_{1i}$ and $\varepsilon_{2i}$ adhere to a trivariate normal distribution with a mean of zero, and the covariance matrix is defined as follows:

(18) $$\mathrm{Cov}\left(\text{i},\varepsilon_{1}\text{and}\varepsilon_{2}\right)=\{\begin{array}{c} \delta 2\delta_{1}\delta_{2}\\ \delta_{1}\delta_{1}^{2}.\\ \delta_{2}.\delta_{2}^{2} \end{array}\text{|}$$

where

The variance of the error terms in the selection equation and two-production commands 1 and 2 are denoted by δ2; $\delta_{1}^{2}$; and $\delta_{2}^{2}$.

The covariance of the selection equation error term (${\;}_{i}$) and the production regimes 1 ($\varepsilon_{1i}$) and 2 ($\varepsilon_{2i}$) is respectively $\delta_{1}$ and $\delta_{2}$. The dot (.) shows that the outcomes of commands 1 and 2 cannot be simultaneously observed for a farmer, and hence, covariance is not present.^45^ In the presence of selection bias, the expectations of the error terms for the two regime equations are different from zero.

(19) $$E[\varepsilon_{1i}|A_{i}=1=\delta_{1}\frac{\emptyset \left(Z_{\mathrm{i\alpha}}\right)}{\Phi \left(Z_{\mathrm{i\alpha}}\right)}=\delta_{1}\lambda_{1i,}$$

(20) $$E[\varepsilon_{2i}|A_{i}=0=-\delta_{2}\frac{\emptyset \left(Z_{\mathrm{i\alpha}}\right)}{1-\Phi \left(Z_{\mathrm{i\alpha}}\right)}=\delta_{2}\lambda_{2i,}$$

where

∅(.) where denotes the standard normal probability distribution.

Φ(.) where denotes the standard normal cumulative distribution.

$\lambda_{1i}$ and $\lambda_{2i}$ are interpreted as inverse Mills ratios incorporated into the production right side equations to capture any selection bias.^46^ The correlation coefficients between the error terms of the production and selection equations were presented.

(21) $$\rho_{1}=\frac{\delta_{1}^{2}}{\delta \delta_{1}}$$

(22) $$\rho_{2}=\frac{\delta_{2}^{2}}{\delta 2}$$

The significance of the estimated covariances of $\rho_{1}$and $\rho_{2}$ reflect that the decision to adopt IMVs, crop yield, and farm returns are correlated, which rejects the null hypothesis of sample selectivity bias. This highlights the importance of the endogenous switching model. In this regard, the full information maximum likelihood estimate provides an efficient ESR output, simultaneously estimating both the selection and production equations. This is higher than the two-step estimators, which are inefficient for deriving standard errors.

The treatment effect of adaptation strategies

This study estimates the effect of adopted IMVs on productivity by employing an endogenous regression model, where adopters are regarded as the treatment group ($A_{i}$ = 1), and their counterfactual is estimated. The observed outcomes for both adapters and non-adapters are outlined below:

(23) $$\mathrm{AdoptersE}[y_{1i}|A_{i}=1]=X_{1i}\beta_{1}+\delta_{1}\lambda_{1i,}$$

(24) $$\mathrm{Non}-\mathrm{adopterE}[y_{2i}|A_{i}=0]=X_{2i}\beta_{2}+\delta_{2}\lambda_{2i,}$$

Similarly, the equation for the counterfactual yield and farm returns of both adapters and non-adapters is as follows:

(25) $$\mathrm{AdopterscounterfactualE}[y_{2i}|A_{i}=0]=X_{1i}\beta_{2}+\delta_{2}\lambda_{1i,}$$

Non-adopters’ counterfactual

(26) $$E[y|A_{i}=0]=X_{2i}\beta_{1}+\delta_{1}\lambda_{2i,}$$

Then the average treated impact of crop yield and farm returns is computed as:

$$\mathrm{ATT}=E\left[y_{1i}\left|A_{i}=1]-E[y_{2i}\right|A_{i}=1\right]=X_{1i}\left(\beta_{1}-\beta_{2}\right)+\left(\delta_{1}-\delta_{2}\right)\lambda_{1i,}$$

The predicted IMVs for crop yield and farm returns for non-adapters (untreated) are:

(27) $$\mathrm{ATU}=E\left[y_{1i}\left|A_{i}=0]-E[y_{2i}\right|A_{i}=0\right]=X_{2i}\left(\beta_{1}-\beta_{2}\right)+\left(\delta_{1}-\delta_{2}\right)\lambda_{2i,}$$

where

ATT – represents the average treatment for the treated (adapters), and ATU – represents the untreated (non-adopters) treatment. The validity of the ESR requires an exclusion restriction that is correlated with adoption, while it does not play a role in the productivity of small-scale crop farmers. Therefore, this study utilized a set of variables as selection instruments, comprising climate information, farm knowledge, attitudes, resource endowment, and distance to the market. These variables are deemed instrumental, as they are crucial factors influencing the decision to adopt IMV, as researchers argue. Nevertheless, these variables did not directly dictate farmers’ productivity levels. The validity of the instruments in the Endogenous Switching Regression (ESR) model was empirically assessed. The initial test employs a logit model to adopt the IMVs and a stochastic production frontier to estimate maize productivity, incorporating both instruments and additional variables. These instruments were collectively confirmed to be robust predictors of adaptation. A distortion test was conducted to ascertain whether the instruments significantly influenced the production process. This investigation indirectly verifies whether the instruments correlate with unobservable factors. The test affirms that the instruments do not collectively and significantly influence productivity among non-adopters.

## Data

Table 1 illustrates the data collected from the smallholder maize farmers.

**Table 1.** Data collected from maize farmers.

Variables	Variable description	Expected sign
**Adoption outcome variables**	**Outcome indicator**	
Binary adoption variable	Dummy = 1 for IMV adopters, 0 otherwise	
Efficiency	Technical efficiency	
Maize productivity	Output per hectare	
**Factor inputs**		
Area under IMV	The total area allocated for IMV cultivation	±
Labour endowment (Ɩ): Family labour	Family members working on maize farm (man-days)	+
Quantity of pesticides	Quantity of pesticides in liters	+
Quantity of fertilizer	Quantity of fertilizer in kilograms	+
Quantity of seed	Quantity of seed in kilograms	+
Total agrochemicals	Quantity of agrochemicals in liters	-
**Household characteristics**		
Sex	Dummy =1 if a farmer is the male household head, 0 otherwise	±
Age	Age of the household head (years)	+
Household monthly income	Total household monthly income (actual amount)	+
Family size	Total family size (in adult-equivalent)	±
Years spent in school	Total number of years of schooling of the household head (years)	±
Asset ownership	The initial value of assets owned by the household	±
Off-farm employment	Dummy = 1 if the farmer participates in off-farm income-generating activities; 0 otherwise	+
**Environmental and Agro-ecologic characteristics**		
Land quality	Land quality index (1= best, … , 9 = worst)	±
Pest occurrence	Equal to 1 if the pest occurred in maize farm in the previous season; 0 otherwise	-
Rainfall shortage (drought)	Dummy = 1 if the household experienced a rainfall shortage in previous production season; 0 otherwise	-
**Institutional characteristics**		
Extension visits	Dummy = 1 if farmer accessed extension service; 0 otherwise	±
Access to credit	Dummy = 1 if farmer accessed credit; 0 otherwise	+
Member of farm organization	Dummy = 1 if the farmer is a member of a farm organization; 0 otherwise	+
Access to improved seed	Dummy = 1 if the farmer has access to IMV seed in the village; 0 otherwise	+
Market access	Dummy = 1 if a village has access to the primary market	+
**Farm characteristics**		
Farm size	The actual size of the farm (actual hectares)	+
Fertile soil	Dummy = 1 if the soil is fertile; 0 otherwise	±
Weeding	Dummy= 1 if the farmer’s frequency of weeding	-

## Results and Discussion

Descriptive statistics for the sampled farmers are provided in Table 2. The data revealed that 62% of the maize farmers are male, indicating a male-dominated sample with some representation of female farmers. These results align with past studies, which indicate that being a male farmer makes it easier to access information and adopt new agricultural techniques than being a female farmer.^47,48^ The average age of farmers is 53 years, suggesting a mature farming population with significant variation in age. Older populations may have different needs and preferences than younger populations.^49^ The farmer’s age was also used as a proxy for farm experience, and these results revealed that the farmers were more experienced, which helped them adopt the IMVs. Farmers typically manage 4-hectare farms and have a household monthly income averaging ZAR 3,562.13, although income levels vary widely, reflecting substantial economic disparity. This is the case for household monthly income, as most farmers rely on different government social security and farm returns, which results in extensive inequalities.^50,51^ Household monthly income levels were crucial and influenced farmers’ purchasing power and investment in farm inputs, especially IMV purchases. The average family size is four members per household, with variability suggesting that family sizes range from small to relatively large. This was also used as a proxy for family labor in the study. Farmers spend 10 years of schooling (according to the South African education system, secondary education), but this is highly variable because it means that farmers can read and interpret agricultural information. Most farmers (72%) face rainfall shortages due to prolonged drought experiences in the province, and 61% participate in non-farm activities, indicating common agricultural and economic challenges. Access to credit is limited to 25% of farmers, and pest problems are prevalent, with an average pest occurrence of 54%. The low access to credit limited farmers’ ability to improve their operations, as some farmers could not afford the IMVs, which led them not to adopt the maize varieties in their farms. Prolonged drought leads to pest occurrence, affecting crop yields and farm productivity, prompting farmers to adopt new seed varieties that can withstand changing environments. These results align with research that found rainfall shortages and drought impact farmers’ productivity.^14^ Most farmers generally perceived soil quality as good, with a mean average score of 0.682, which reflects that most farms have good and fertile soil that favors crop farming. Approximately 66% of farmers are members of farm organizations, suggesting that membership is relatively standard among this group. Membership in farm organizations provided farmers with support, resources, and networking opportunities, which led to the adoption and implementation of IMVs on their farms. Access to extension services was high (with 65%), indicating that a significant portion of farmers have access to extension services, which played a crucial role in smallholder farmers’ adoption of IMVs in the study. These results concur with past studies that found that access to extension services was essential in adopting maize-improved varieties in the province.^39,52^ The study reveals that an average of 0.613 farmers have access to and planted a specific type of maize variety on their farms. The mean distance to markets is 30.23 kilometers, indicating variability in respondents’ distance from their markets. Distance to markets affects transportation costs, market access, and overall efficiency, which results in lower farm returns owing to the higher costs incurred. The 79% suggests that IMVs seeds are relatively available in the market, and farmers can access them. The availability of IMVs significantly affects agricultural productivity and farm returns. However, the variability in the availability of these seeds may reflect regional differences in supply and demand. Smallholder farmers also specified that, on average, they have herd sizes 15, which also assists in providing organic fertilizer for maize production.

**Table 2.** Demographic characteristics of maize farmers and summary of variables used in the models.

Variable	Mean	Std Deviation
**Variables used in ESR and Logit Model**		
Sex (male)	0.623	0.353
Access and planted Variety type	0.613	0.312
Age	53.421	16.942
Family size	4.176	2.508
Years spent in school	10.436	9.785
Farm size	3.542	2.587
Household monthly income	3 562.13	4530.791
Soil quality	0.682	0.454
Access to credit	0.251	0.358
Participation non-farm activities	0.605	0.498
Access to extension services	0.650	0.247
Member of farm organization	0.663	0.269
Pest occurrence	0.541	0.351
Rainfall shortage in the area	0.721	0.436
Distance to markets	30.23	19.28
IMVs seeds availability in the market	0.7893	0.328
Herd size	15.356	13.614
**Variables used in the SPF models**		
Output	659.552	861.317
Labour	386.596	370.592
Pesticides	5.094	3.481
Fertilizer	64.199	104.789
Seed	8.663	10.063
Agrochemicals	2.098	2.944

Regarding inputs for maize production, the results found that, on average, maize output was 659.55 units with considerable variability. At the same time, the average labor input (family labor) is 386.596 units, suggesting significant differences in labor use across farms. They found that, on average, maize farmers used 5.094 units of pesticides, and the average use of agrochemicals was 2.098 units. These results suggest variability in the use of these inputs by maize farmers. Maize farmers use an average of 8.663 seed units, while fertilizer use is 64.199 units, revealing a wide range of fertilizer application practices among maize farmers. The average maize output is 659.552 units, indicating substantial variability in maize yields among farmers. These results reveal that inputs for maize production, including labor, pesticides, fertilizers, seeds, and agrochemicals, exhibit significant variation, reflecting diverse farming practices and productivity levels across the sampled farmers.^52,53^

### Types of Improved Maize Seed Adopted by Smallholder Farmers

**Figure 2.**
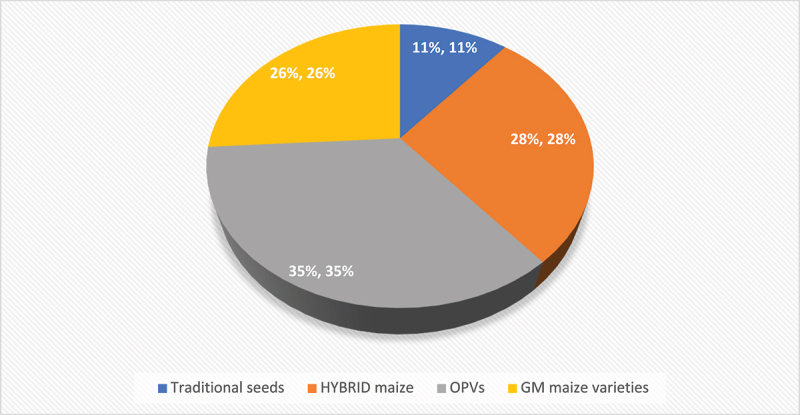
Distribution of improved maize seed types.

Figure 2 illustrates the distribution of improved maize seed types among smallholder farmers, revealing important insights into their preferences, access to technology, and potential impact on agricultural productivity. Traditional seeds, used by only 11% of farmers, highlight the dwindling reliance on historical varieties. This low adoption rate suggests a shift toward modern, improved seed technologies due to changing environmental conditions, which do not favor traditional seeds as they cannot withstand the changing climate. However, the continued use of traditional seeds points to cultural preferences, lower costs, and limited access to improved varieties. In contrast, open-pollinated varieties (OPVs) (such as improved (Sahara) and unimproved (Landrace)) are the most commonly adopted seed type at 35%, indicating that their balance of cost and performance appeals to many smallholder farmers. Improved OPVs offer better yields and resilience, yet unimproved varieties may still be used for local adaptation or at a lower cost. These results emphasize the sentiments of past studies that found that OPVs are the most adopted IMVs in the province because of their accessibility and affordability to farmers.^50,54,55^ Hybrid maize, used by 28% of farmers, reflects a growing recognition of its productivity benefits, although barriers such as cost and availability might limit its broader adoption. Genetically Modified (GM) maize varieties (such as Roundup ready and Stacked bt and ht genes), adopted by 26% of farmers, show a significant openness to advanced technology to overcome specific challenges such as pests and weeds.

To fully transition to modern seeds, it is crucial to understand and address these factors, ensuring that improvements are tailored to local conditions and farmers’ preferences. To further increase the adoption of hybrids and GM varieties, strategies should focus on enhancing accessibility, reducing costs, and providing comprehensive education on their benefits while addressing regulatory or market acceptance issues.

## Benefits from Adopted IMVs by Smallholder Farmers

**Figure 3.**
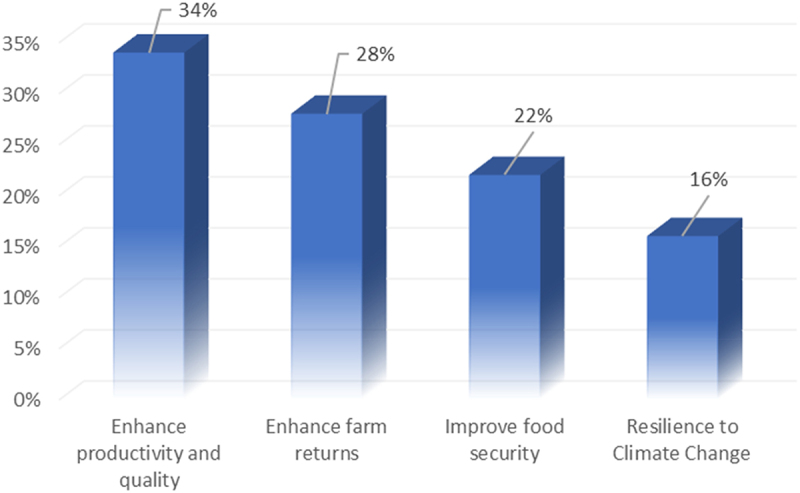
Benefits of adopted IMVs among smallholder farmers.

Figure 3 below illustrates the benefits of the adopted IMVs by smallholder farmers. Adopting improved maize varieties (IMVs) provides significant benefits to smallholder farmers. IMVs boost maize productivity and quality by 34%, offering higher yields per hectare and more efficient use of resources such as soil, water, and sunlight, even under challenging conditions. They feature faster growth, better tillering, uniform maturation, streamlined harvesting, and reduced losses. These varieties also enhance grain quality with better nutritional value, taste, and consistency and improve storage, minimizing post-harvest losses. As a result, farmers experience increases farm productivity and food security. Additionally, IMVs enhance farm returns by 28% through higher yields and better-quality maize, which directly boost income and economic stability. They reduce costs by being more resistant to pests and diseases, thus lowering the need for costly inputs. This improved productivity and quality enhance market competitiveness, leading to better pricing and market access and allowing farmers to invest in further improvements, thereby supporting long-term economic growth and stability.

IMVs are crucial for enhancing food security (22%) by ensuring a stable and reliable food supply and improving nutritional quality. Higher yields from these varieties lead to consistent availability of maize, which is essential in the province where maize is a staple food, helping to stabilize the food supply and reduce vulnerability to shortages caused by market fluctuations or adverse weather. Additionally, these varieties offer better nutritional profiles, address malnutrition, and ensure that essential vitamins and minerals are more readily available. This increased production also allows smallholder farmers to better meet their food needs, reduce dependence on external sources, and enhance household food security.

Finally, IMVs significantly bolster resilience to climate change (16%) by enhancing adaptation to extreme conditions and promoting sustainable farming practices among smallholder farmers. These varieties are often bred for drought tolerance, enabling them to endure periods of low water availability, which is becoming increasingly crucial as climate change exacerbates drought frequency and severity. They also feature enhanced resistance to diseases and pests, helping to mitigate environmental stressors on yield and quality. Additionally, improved maize varieties typically require less intensive soil management, soil health preservation, and erosion reduction. Their adoption encourages implementing climate-smart agricultural practices such as conservation tillage and better water management, further enhancing agricultural resilience and sustainability.

### Factors Influencing the Adoption of IMVs by Smallholder Maize Farmers

Table 3 illustrates the factors influencing IMV adoption by smallholder farmers in the study area. For this study, model fit is assessed using the following statistics: −2 Log Likelihood (266.55107), Chi-Square (159.286 with 13 degrees of freedom), Prob<Chi-Square (0.000), and Pseudo R^2^ (0.71827). The −2 Log Likelihood measures how well the model fits the data, with lower values indicating a better fit and 0 being the theoretical best fit. The Chi-Square statistic tests the overall significance of the model by comparing it to a baseline model with no predictors. A Chi-Square value of 159.286, with a p-value of 0.000, indicates that the model’s predictors significantly improve the fit, showing that the model is statistically significant. A Pseudo R^2^ value of 0.71827 indicates that the model explains a substantial proportion of the variance in the dependent variable, reflecting a high level of fit. A Pseudo R^2^ value of 72% indicates a strong model fit. These measures suggest that the logit regression model effectively captures the relationships between the predictors and outcome variables, demonstrating a strong fit for the data.

**Table 3.** Factors influencing the adoption of IMVs by smallholder farmers.

Variable	Coeff	Std Error	t-value	Marginal Effects
Years spent in school	1.458***	0.467	3.122	0.028
Farm size	0.535**	0.207	2.585	0.039
Distance to markets	−0.349**	0.053	−6.585	0.032
Age	0.615**	0.242	2.541	0.025
Family size	−0.482***	0.196	−2.459	0.027
Membership in farm organization	0.895**	0.472	1.896	0.043
Access to extension services	1.690***	0.786	2.150	0.045
Seed availability	1.646 ***	0.735	2.239	0.053
Access to credit	1.861	0.680	2.737	0.048
const	1.943**	0.773	2.51	
−2 Log likelihood 266.55107	Prob<X^2^ 0.000	Pseudo R^2^ .71827	Chi-square (13) 159.286	Obs = 150

*** refers to significant at 1%, ** refers to significant at 5%.

Table 3 presents a regression analysis of the factors influencing smallholder farmers’ adoption of improved maize varieties (IMVs). The results highlight several key factors influencing smallholder farmers’ adoption of improved maize varieties. Education, farm size, and access to resources such as extension services, seed availability, and credit significantly increase the likelihood of adoption. In contrast, distance to markets and family size negatively impact adoption. These findings underscore the importance of education, resource access, and organizational support in facilitating the adoption of new agricultural technologies.

Years spent in school had a positive and statistically significant coefficient at the 1% level, indicating a robust relationship between educational attainment and adoption of improved maize varieties (IMVs). This implies that a farmer’s additional years spent in school will significantly enhance the likelihood of adopting IMVs. A marginal effect of 0.028 implies that for each additional year of schooling, the probability of adopting improved maize varieties increases by 2.8%. This indicates a positive effect of education on the likelihood of the outcome. This shows that higher education positively influences adoption due to increased awareness, knowledge, and access to information about agricultural practices and technologies. These results align with past studies that education plays an important role in farmers’ decision-making and in adopting improved maize varieties to enhance maize productivity.^39,51,54,56^ Specifically, this suggests that with every additional year of education, the probability of a farmer adopting IMVs increases by 2.8%. This substantial effect highlights that more educated farmers will likely be better equipped to comprehend and implement new agricultural technologies due to improved problem-solving skills, better access to information, and enhanced capacity to evaluate and manage innovations. Consequently, investing in education can be a powerful strategy to increase the adoption of advanced agricultural practices among smallholder farmers, foster agricultural development, and improve productivity.

Farm size had a positive and statistically significant coefficient at the 5% level, revealing that larger farm sizes significantly increased the likelihood of adopting improved maize varieties (IMVs). This implies that the farmer’s additional hectare of farm size increases the probability of adopting IMVs. The marginal effect of 0.039 means that as farm size increases, the probability of adopting improved maize varieties increases by 3.9%. Larger farm sizes allow farmers more resources and capacity to invest in improved varieties, enhancing their productivity. These results emphasize that farm size creates favorable conditions for applying new agricultural techniques and technology to enhance productivity and farm returns.^57^ This means that for every additional unit increase in farm size, the likelihood of adopting IMVs increased by approximately 3.9%. The increased adoption among larger farms can be attributed to their more excellent resources, such as access to capital, labor, and technology, which facilitate investment in new agricultural innovations. Larger farms are also better positioned to manage the financial and operational risks of adopting new technologies, such as improved maize varieties. Thus, the capacity to absorb potential risks and invest more in agricultural advancements contributes to the higher adoption rates of IMVs on larger farms.

Distance in markets was negative and statistically significant at the 5% level, indicating a robust inverse relationship between distance to the market and the adoption of improved maize varieties (IMVs). This suggests that increasing the distance to markets by 1 additional kilometer will decrease the likelihood of adopting IMVs. The marginal effect of 0.032 implies that the probability of adopting improved maize varieties decreases by 3.2% for each additional kilometer in the distance to the market. This suggests that farmers far from markets face higher transaction costs, limiting their ability to access inputs like improved seeds and fertilizers. This negative effect is likely to be attributable to several factors. An increased distance to markets can lead to higher transaction costs for transporting inputs and harvested produce, making it less economically viable to adopt new technologies. Additionally, farmers farther from markets may face challenges accessing timely and accurate market information, which is crucial for making informed decisions regarding technology adoption. These results agree with research conducted in the past, which states that most farmers are situated far from town, which reduces their adoption rate to a minimum because of the high transaction costs involved and smallholder farmers having limited financial support.^56,58^ These factors collectively contribute to the lower adoption rates of IMVs among farmers farther from markets, underscoring the importance of improving market access and infrastructure to support agricultural innovation.

Age had a positive coefficient and was statistically significant at the 5% level, highlighting a positive and statistically significant relationship with the adoption of improved maize varieties (IMVs). This indicates that an additional year in the farmers’ age increases the likelihood of adopting IMVs. This is because older farmers must have accumulated extensive farming experience and possess a deeper understanding of agricultural practices and a higher confidence level in evaluating new technologies. Additionally, older farmers might have a greater tolerance for the risks of adopting innovations, possibly because of their established farming systems and financial stability. These results align with previous research that older and experienced farmers readily adopt new technologies to enhance productivity, increase household food, and sell surpluses to generate income.^10,59^ The marginal effect of 0.025 means that for each year of age, the probability of adopting improved maize varieties increases by 2.5%. This indicates that older farmers with more farm experience may be more willing to invest in or adopt new technologies based on their accumulated knowledge. This moderate effect suggests that while age influences adoption, it does so relatively incrementally. Thus, age, experience, and risk tolerance enhance the adoption of advanced agricultural practices. However, it is only one of several factors influencing the decision to adopt IMVs.

Family size had a negative coefficient and was statistically significant at the 1% level, indicating a negative and statistically significant impact of adopting improved maize varieties (IMVs). This implies that increasing the family size by 1 person will reduce the likelihood of adopting IMVs. This negative effect occurs because larger families may face increased labor demands and resource constraints that limit their capacity to invest in and manage new agricultural technologies. For instance, the additional labor needed to support a more enormous household might diver from farming activities, or increased household expenses could reduce the financial resources available for adopting new technologies. These results concur with past research that family size adversely affects adopting improved maize varieties.^56,60^ As a result, they compete with IMVs resources, as they need such resources (money) to purchase the basic needs they require for survival. However, the results contradict the literature, where family size positively affected the adoption of IMVs.^57^ The marginal effect of 0.027 means that for each additional family member, the probability of adopting improved maize varieties decreases by 2.7%. Larger families are more resource-constrained and have less flexibility to invest in improved technologies due to competing financial and labor needs. This reflects how the increased burden of managing a more enormous household may lead to a lower propensity to take on risks and costs associated with adopting improved maize varieties. Thus, family size significantly shapes farmers’ decisions to adopt new agricultural innovations through its influence on labor and resource allocation.

Membership in farm organizations had a positive coefficient and was statistically significant at the 5% level, highlighting a positive and statistically significant impact on adopting improved maize varieties (IMVs). This implies that a unit increase of 1% in being a member of a farm organization significantly increases the likelihood of adopting IMVs. The positive effect is likely due to the various benefits offered by farm organizations, including improved access to crucial information, technical support, and resources that facilitate the adoption of new agricultural technologies. Members of these organizations often benefit from shared knowledge, collective purchasing power for inputs, and access to training and extension services, all of which can enhance their capacity to implement and manage innovations such as IMVs. These results concur with the previous research that being a member of a farm organization increases the probability and likelihood of adopting improved maize varieties by interacting with neighboring farmers who provide better access to improved agricultural techniques and technology as well as the training provided by the organization.^14,39,61^ The marginal effect of 0.043 means that 1% induces an increase in farm organization membership, increasing the probability of adopting improved maize varieties by 4.3%. Membership in farm organizations likely provides better access to information, extension services, and collective action, making it easier for farmers to adopt new technologies. This substantial impact underscores the role of farm organizations in bridging gaps between farmers and technology providers, thus promoting the diffusion of improved agricultural practices and contributing to more widespread adoption of innovations.

Access to extension services has a positive coefficient and is statistically significant at the 5% level, signifying a positive and statistically significant effect on adopting improved maize varieties (IMVs). This implies that a unit increase of 1% in access to extension services and visits by extension agents significantly increases the likelihood of IMV adoption. The high value underscores the critical role that extension services play in facilitating the adoption process. Extension services provide farmers with essential technical support, updated information, and practical advice on the effective implementation of new agricultural technologies. This guidance helps farmers understand the benefits of IMVs, how to manage them, and how to overcome potential challenges. These results underscore that extension services increase awareness and access to new agricultural technologies to improve farmers’ productivity and farm returns.^10^ The marginal effect of 0.045 means that a 1% unit increase in access to extension services increases the probability of adopting improved maize varieties by 4.5%. Extension services provide farmers with critical knowledge and skills, directly influencing their adoption decisions by improving their understanding of using improved maize varieties effectively. This substantial effect reflects the importance of extension services in bridging the gap between technology developers and farmers, thereby enhancing the dissemination and successful adoption of improved agricultural practices. The findings highlight that strengthening extension services can be a key strategy for promoting innovation uptake and improving agricultural productivity.

Seed availability had a positive coefficient and was statistically significant at the 1% level, demonstrating a positive and statistically significant effect of adopting improved maize varieties (IMVs). This suggests that a unit increase of 1% in the availability of improved seeds enhances the likelihood of IMV adoption. When farmers have easier access to high-quality seeds, they are more likely to incorporate them into their farming practices, leading to higher adoption rates. The marginal effect of 0.053 means that an induced unit increase of 1% better seed availability increases the probability of adopting improved maize varieties by 5.3%. Availability of high-quality seeds is a direct enabler of adoption, and improved access to seeds makes it easier for farmers to adopt new technologies. This significant impact underscores the essential role of seed accessibility in facilitating the adoption process. Regardless of other supporting factors, farmers may struggle to implement new technologies effectively without a reliable supply of improved seeds. Therefore, improving seed distribution channels and ensuring consistent availability of quality seeds are crucial for promoting the widespread adoption of agricultural innovations and enhancing overall productivity in smallholder farming systems.

Access to credit has a positive coefficient and is statistically significant at the 1% level, indicating a positive and statistically significant effect on adopting improved maize varieties (IMVs). This implies that a unit increase of 1% in access to credit significantly enhances farmers’ likelihood of investing in IMVs. The availability of financial resources through credit allows farmers to cover the upfront costs associated with adopting new technologies, such as purchasing improved seeds, fertilizers, and other necessary inputs. The marginal effect of 0.048 means that a 1% unit-induced access to credit increases the probability of adopting improved maize varieties by 4.8%. Access to financial resources allows farmers to invest in inputs like improved seeds, fertilizers, and irrigation systems, thereby facilitating adoption. This substantial effect highlights that financial constraints can be a major barrier to adopting new agricultural practices. These results highlights that having access to credit enhances the adoption of IMVs by enabling farmers to purchase the required farm inputs that increase productivity.^14^ By providing access to credit, farmers can overcome financial hurdles and invest in innovations that promise to improve their productivity and farm income. Consequently, improving access to credit is essential to enable smallholder farmers to embrace advanced technologies and enhance their agricultural outcomes.

### Technical Efficiency of Smallholder Maize Farmers Adopted IMVS in Their Farms

This section presents and discusses the SFA estimation of technical efficiency scores. Table 4 presents the Maximum Likelihood estimates of the Cobb-Douglas stochastic production frontier parameters. The stochastic production frontier parameters’ maximum likelihood estimates (MLE) reveal crucial insights into IMVs production dynamics among smallholder farmers. The Sigma-Square (δ^2^ estimate is 2.6554, with a standard error of 0.7462 and a t-statistic of 3.559, indicating substantial overall variance in the model’s error term. This high value reflects considerable variability in production, encompassing both inefficiencies and random shocks that are not explained by the model. Gamma (γ) is estimated at 0.9931 with an exceptionally low standard error of 0.0024 and a t-statistic of 410.175, suggesting that nearly all the output variability is attributed to differences in inefficiency rather than random noise. This near-unity γ value underscores the dominance of inefficiency as a factor in the production variation. The significance of (γ) indicates that the technical inefficiency effects are significant in determining the level and variability of maize production. From the estimated value of (γ), it is observed that only about one percent of random variation in maize production is attributed to random shocks that are out of the farmers’ control. Additionally, the log-likelihood function value of −84.59 and the likelihood ratio test statistic of 298.98 further support the inclusion of inefficiency effects in the model, as the high LR test value signifies a statistically significant improvement over a model that excludes inefficiency effects. These results indicate that inefficiency is the primary source of variation in maize production among smallholder farmers, overshadowing random shocks and measurement errors, and affirm the model’s effectiveness in capturing these inefficiencies.

**Table 4.** Maximum likelihood estimates (MLE) for Cobb-Douglas stochastic frontier production function parameters for adopted IMVs maize production on smallholder farmers.

Variable	Parameter	Coef	Std Error	t-ratio
Constant	$\beta_{0}$	0.691***	0.047	14.702
Ln Farm size	$\beta_{1}$	0.637***	0.061	10.442
Ln Seed	$\beta_{2}$	0.664***	0.154	4.312
Ln Fertilizer	$\beta_{3}$	0.539***	0.071	7.592
Ln Labour	$\beta_{4}$	−0.343**	0.197	−1.741
Ln Pesticides	$\beta_{5}$	0.489**	0,185	2.643
Ln Agrochemicals	$\beta_{6}$	0.074	0,067	1.104
**Diagnostic Statistics**				
Sigma-Square	$\delta^{2}=\delta_{\mu }^{2}+\delta_{v}^{2}$	2.6554***	0.7462	3.559
Gamma (γ)	$\gamma =\delta_{\mu }^{2}/\delta^{2}$	0.9931***	0.0024	410.175
LR function	−84.59			
LR test	298.98			

*** refers to significant at 1%, ** refers to significant at 5%.

The coefficient for Ln Farm Size (β_1), valued at 0.637 and significant at the 1% level, indicates a robust positive relationship between farm size and maize production among smallholder farmers adopting improved maize varieties (IMVs). This suggests that, as the area of land under cultivation increases by an additional hectare, so does the output of maize, highlighting the economies of scale inherent in agricultural production. This finding emphasizes the critical role of farm size as a determinant of productivity. Larger farms may benefit from greater resource allocation, enhanced mechanization possibilities, and improved management practices, collectively contributing to higher yields. Additionally, larger land areas allow for better crop rotation and diversification strategies, potentially increasing overall resilience to pests and climate variability. Conversely, smallholder farmers with limited land may face constraints that hinder their ability to effectively optimize production inputs. This relationship emphasizes the importance of land access and size in agricultural policy and support systems to improve food security and farmers’ livelihoods.

The coefficient for Ln Seed (β_2), at 0.664 at a 10% significance level, reveals a strong positive correlation between seed inputs and maize production among smallholder farmers utilizing improved maize varieties (IMVs). This finding suggests that adopting high-quality, improved seed varieties enhances agricultural productivity. Specifically, the coefficient indicates that a 1% increase in seed use (IMVs) is associated with a more than proportional increase in maize yield. This can be attributed to the superior genetic traits of the improved seeds, such as higher disease resistance, better drought tolerance, and increased nutrient efficiency. This reinforces the idea that access to quality seeds is not merely an input but a fundamental driver of agricultural success.

The coefficient for Ln Fertilizer (β_3), recorded at 0.539 and significant at the 15 level, highlights a significant positive impact of fertilizer use on maize production among smallholder farmers cultivating improved maize varieties (IMVs). These findings highlight the essential role of fertilizer in enhancing crop yields by providing the necessary nutrients that are often deficient in the soil, particularly in regions where soil fertility has been depleted due to intensive agricultural practices or inadequate soil management. The positive coefficient suggests that as farmers increase their investment in fertilizers, they can expect a corresponding increase in maize output, emphasizing the importance of proper nutrient management for optimizing production. This emphasizes the need for policies and initiatives that improve farmers’ access to quality fertilizers, such as subsidies, training on optimal application rates, and integrated soil fertility management practices.

The coefficient for Ln Labour (β_4), recorded at −0.343 and significant at the 5% level, presents an intriguing insight into the relationship between labor input and maize production among smallholder farmers utilizing improved maize varieties (IMVs). The negative coefficient indicates that, contrary to expectations, an increase in labor does not translate into a proportional increase in production; rather, it suggests that additional labor may reduce overall productivity. This phenomenon could be attributed to several factors, including inefficiency in labor utilization, such as overstaffing during peak periods or a lack of skilled labor, leading to disorganization and reduced output. Furthermore, diminishing returns may be at play, where the marginal productivity of additional labor decreases as more labor is added, especially if other inputs (such as capital or technology) are not scaled up.

The coefficient for Ln Pesticides (β_5), valued at 0.489 and significant at the 5% level, reveals a noteworthy positive relationship between pesticide use and maize production among smallholder farmers utilizing improved maize varieties (IMVs). This indicates that effective pest management practices, facilitated by the use of pesticides significantly enhance crop yields. The positive coefficient suggests that farmers can expect a corresponding increase in maize output as they increase their application of pesticides, primarily due to the reduction in crop losses associated with pest infestations. The significance of this variable underscores the critical role that pest control plays in achieving optimal agricultural productivity. Effective pest management protects the immediate yields and contributes to the overall health of crops, reducing stress and improving resilience against adverse conditions. This finding emphasizes the importance of education and access to appropriate pest control measures for smallholder farmers to ensure they can adopt integrated pest management strategies that balance pesticide use with sustainable agricultural practices.

## Analysis of Technical Inefficiency Model Parameters

Hypothesis testing verified that technical inefficiency effects are evident in the Stochastic Frontier Analysis (SFA) model. Table 5 shows the MLE results of the technical inefficiency model for the IMVs of the maize farmers. Considering these inefficiencies, enhancing farmers’ technical efficiency by exploring the factors contributing to technical inefficiency is crucial. Consequently, this section presents and analyses the parameters of the technical inefficiency model specifically for smallholder maize farmers.

**Table 5.** Maximum likelihood estimation results of technical inefficiency model variables.

Variable	Parameter	Coef	Std Error	t-ratio
Constant	$\alpha_{0}$	−1.594***	0.324	4.919
Age	$\alpha_{1}$	−0.598**	0.154	3.883
Family size	$\alpha_{2}$	−1.416***	0.552	2.565
Years spent in school	$\alpha_{3}$	−0.628**	0.251	2.502
Access to extension services	$\alpha_{4}$	−0.804**	0.295	2.725
Access to credit	$\alpha_{5}$	−0.476***	0.147	3.238
Herd size	$\alpha_{6}$	0.628**	0.251	2.502
Off-farm income	$\alpha_{7}$	0.887**	0.315	2.816
Distance to market	$\alpha_{8}$	−0.952***	0.301	3.163
Frequency of weeding	$\alpha_{9}$	−1.622**	0.798	2.032
Seed type	$\alpha_{10}$	−0.649**	0.275	2.360

***refers to significant at 1%, ** refers to significant at 5%.

Smallholder farmers’ maximum likelihood estimation results of the technical inefficiency model for adopted Improved Maize Varieties (IMVs) provide insightful parameters regarding the factors that affect technical inefficiency. The constant term in the model, with a statistically significant negative coefficient, indicates that, in the absence of other variables, the baseline level of technical inefficiency is relatively high. This suggests that inefficiency in maize production is substantial without considering other factors.

Age had a negative coefficient and was statistically significant at a 5% level (α₁ = −0.598), indicating that older farmers exhibit lower levels of technical inefficiency in their maize production practices than younger farmers. This result suggests that farmers’ inefficiency in utilizing agricultural inputs and practices, such as IMVs, decreases as they age. One plausible explanation for this phenomenon is that older farmers accumulate substantial experience and practical knowledge during their years of farming. These results emphasize that only younger farmers had technical efficiency in production due to their desire to transform smallholder farming into agribusiness and be progressive.^13^ This experiential learning enables older farmers to make more informed decisions, adopt more effective farming techniques, such as IMVs, and manage resources more efficiently than their younger counterparts. Additionally, older farmers may have better skills in handling the challenges of farming, having learned from years of trial and error. However, it is also essential to consider that while experience generally enhances efficiency, very advanced age might eventually introduce limitations, such as reduced physical stamina or slower adoption of new technologies. Overall, this finding highlights the value of farming experience, suggesting that older farmers’ accumulated knowledge contributes significantly to their operational efficiency in maize production by adopting IMVs to enhance productivity.

Family size has a negative and statistically significant coefficient for family size at the 1% level (α₂ = −1.416), revealing that larger family sizes are linked to reduced technical inefficiency in maize production. This association suggests that having a more prominent family can positively contribute to farm efficiency. This is also because of the increased availability of labor, which allows for more intensive and effective management of farming activities such as planting, weeding, and harvesting. Additional family members can share the workload, leading to timely and thorough execution of farm operations, directly enhancing productivity and reducing inefficiencies. These results indicate that the negative effect implies that larger family sizes do not allocate resources to farming and that larger family sizes result in technical efficiency challenges due to labor challenges.^13,62^ Moreover, a more prominent family may provide better decision-making and resource management support as more individuals can contribute to diverse skills and knowledge. This increased labor and support network can improve agricultural practices and technology implementation (IMVs), ultimately improving operational efficiency. Thus, larger family sizes are a significant asset in optimizing maize production processes and mitigating inefficiencies that might arise from labor shortages or inadequate farm management.

Years spent in school had a negative and statistically significant coefficient for years spent at the 5% level (α₃ = −0.628), highlighting a crucial link between education and technical efficiency in maize production. This result suggests that farmers with more years of schooling experienced lower levels of technical inefficiency. The underlying reason for this negative relationship is likely the enhanced capacity of educated farmers to adopt and implement advanced agricultural practices such as IMVs. Education is a significant productivity element because different skills such as farm management and bookkeeping, are often taught. Education equips farmers with improved problem-solving skills, better understanding of modern farming techniques, and the ability to interpret and apply technical information more effectively. These findings emphasize that educated farmers are less technically efficient than full-time farmers as they spend most of their time investing in non-agricultural activities and only part-time farming.^13,63^ This knowledge facilitates efficient farming methods, optimization of resource use, and the adoption of innovative technologies that can significantly enhance productivity. Additionally, educated farmers are often better at managing farm operations, analyzing market conditions, and making informed decisions, which collectively contribute to reducing inefficiencies. Thus, investing in educational opportunities for farmers can be a powerful strategy to improve agricultural efficiency and boost overall production outcomes. These results contradict with literature that found that education positively influenced productivity and technical efficiency.^64,65^

Access to extension services had a negative and statistically significant coefficient for access to extension services at a 5% level (α₄ = −0.804), underscoring the critical role these extension services play in enhancing agricultural efficiency. This result indicates that maize farmers with access to extension services experienced notably lower levels of technical inefficiency. Extension services are designed to bridge the gap between research and practical applications, by offering farmers essential knowledge, guidance, and support tailored to local agricultural conditions. They provide crucial information on best practices, new technologies, pest management, and crop optimization strategies, which enable farmers to make informed decisions and improve their farming practices. Extension services help farmers increase productivity, reduce waste, and manage resources efficiently by facilitating the adoption of improved techniques and technologies. Consequently, this support directly reduces technical inefficiency, highlighting the importance of robust extension systems for enhancing farm performance and ensuring sustainable agricultural development. These results emphasize that access to extension personnel influences technical efficiency, which is expected to enhance productivity.^10,66^

Access to credit had a negative coefficient and was statistically significant for access to credit at a 1% level (α₅ = −0.476), highlighting the crucial role that financial resources play in reducing technical inefficiency among farmers. This result underscores the fact that access to credit is a key factor in improving agricultural efficiency. When farmers can secure loans or financial assistance, they can invest in high-quality inputs such as seeds, fertilizers, and advanced technologies that are essential for optimizing production. This financial capability allows adopting innovative practices and equipment that enhance operational efficiency and productivity. Furthermore, credit access can provide the necessary funds for expanding farm operations, managing risks, and smoothing out cash flow issues, all of which contribute to more effective farm management. Therefore, facilitating access to credit is vital for empowering farmers to overcome financial constraints and make investments that drive improvements in production efficiency, ultimately leading to reduced technical inefficiency and improved overall farm performance.

Herd size had a positive coefficient and was statistically significant for herd size at the 5% level (α₆ = 0.628), indicating that larger herd sizes are linked to higher technical inefficiency in maize production. This result suggests that as herd size increases, so do inefficiencies in managing and utilizing farm resources. Larger herds can strain available resources, such as feed, water, and labor, leading to challenges in maintaining optimal health and productivity of livestock. In addition, the complexity of managing a larger herd may overwhelm farm operations, resulting in suboptimal practices and reduced efficiency. Overgrazing, increased disease management, and logistical difficulties in feeding and caring for herds can contribute to these inefficiencies.^13^ Consequently, while a larger herd has the potential to increase overall production, it also requires more sophisticated management and resource allocation strategies to avoid inefficiencies that detract from the farm’s overall productivity.

Off-farm income had a positive coefficient and was statistically significant for off-farm income at the 5% level (α₇ = 0.887), revealing a notable association between higher off-farm income and increased technical inefficiency in maize production. This relationship suggests that as farmers earn more from off-farm activities, their technical inefficiency in farming tends to increase. One likely explanation for this phenomenon is that farmers with substantial off-farm incomes may allocate less time and attention to their agricultural operations. Additional financial resources from off-farm activities might reduce the urgency or necessity for efficient farm management, leading to a less rigorous approach to farming tasks such as crop care, resource optimization, and timely interventions. Furthermore, with less dependence on farming for their livelihood, these farmers might not prioritize or invest in enhancing farming practices and technologies. Consequently, the focus and energy diverted to off-farm income opportunities could detract from the efficiency and effectiveness of agricultural activities, resulting in higher technical inefficiency.

Distance to markets had a negative coefficient and was statistically significant for distance to market at a 1% level (α₈ = −0.952), suggesting that a greater distance to market is linked to reduced technical inefficiency in maize production. This counterintuitive result indicates that farmers farther from the markets may experience lower levels of technical inefficiency. One possible explanation is that distance to the market might incentivize farmers to adopt more efficient production practices to offset the higher costs and logistical challenges associated with transporting their produce over long distances. To mitigate the impact of distance, these farmers could invest in better production techniques, optimize resource use, and improve overall farm management to ensure their crops are of high quality and yield. The negative coefficient of distance to the market might also reflect improved market access or infrastructure developments in more remote areas, which can facilitate better market integration and lower transaction costs. Thus, the relationship suggests that, while distance poses challenges, it can also drive farmers to enhance their efficiency and productivity to remain competitive and manage market access costs.

The frequency of weeding had a negative coefficient and was statistically significant for the frequency of weeding at the 5% level (α₉ = −1.622), indicating that a higher frequency of weeding is associated with reduced technical inefficiency in maize production. Although the coefficient is significant, it is not highly negative, suggesting a notable but moderate effect of increased weeding frequency on efficiency. This result underscores the importance of regular weeding as a key practice for effective crop management. Frequent weeding helps to control weed competition for nutrients, water, and sunlight, which can diminish crop yield and quality. By reducing weed pressure, farmers can enhance the growth and productivity of their maize crops, leading to a more efficient use of inputs and ultimately improved yields. This finding implies that increased weeding frequency contributes to greater efficiency, but its impact may be less pronounced than other factors. Nevertheless, consistent and timely weeding is essential to optimize farm productivity and reduce inefficiencies. These results indicate that when more farmers frequently weed their farms, their productivity and technical efficiency are enhanced.^13^

Seed type had a negative coefficient and was statistically significant for seed type at the 5% level (α₁₀ = −0.649), demonstrating that improved seed varieties are associated with reduced technical inefficiency in maize production. This negative coefficient aligns with the expectation that high-quality seeds will enhance production efficiency. Improved seed varieties have been specifically developed to offer better resistance to pests and diseases, higher yield potential, and more efficient use of nutrients and water than traditional seeds. Farmers can achieve better crop performance and higher yields by utilizing these advanced seeds, which directly lowers technical inefficiency. The results highlight the significant impact of seed quality on agricultural productivity, underscoring the importance of adopting improved seed varieties to optimize farm operations and achieve more efficient use of resources. This finding reinforces the importance of investing in and promoting high-quality seeds as a crucial strategy for enhancing agricultural efficiency and productivity.

### Distribution of Efficiency

The distribution of technical efficiency scores among smallholder maize farmers provides a comprehensive overview of their operational performance. The Table 6 below shows the distribution of the efficiency. With a sample of 150 farmers surveyed, the efficiency scores range from a low of 0.10 to a high of 0.91, reflecting a broad spectrum of efficiency levels. The mean efficiency score is 0.74, indicating that, on average, farmers operate at a relatively high level of technical efficiency. However, there is notable variability across different efficiency ranges. A small fraction of farmers (0.31%) fell within the lowest efficiency range (≤0.20), suggesting that extreme inefficiency is rare but present. Conversely, a substantial proportion, 26.6%, score between 0.61 and 0.70, representing the largest single group. This indicates that many farmers are relatively efficient, although not at the highest possible level. The range between 0.41 and 0.50, with 16.0% of farmers, shows a considerable segment of moderately efficient operations.

**Table 6.** Distribution of technical efficiency scores.

Efficiency range	Frequency	Percentage (%)
≤0.20	1	0.31
0.21–0.30	5	2.72
0.31–0.40	16	10.3
0.41–0.50	22	16.0
0.51–0.60	14	8.2
0.61–0.70	37	26.6
0.71–0.80	26	18.4
0.81–0.90	11	6.27
0.91–1.00	18	11.2
**Total**	**150**	**100**
**Efficiency scores**		
Mean	0.74	
Minimum	0.10	
Maximum	0.91	

On the higher end, 18.4% of farmers score between 0.71 and 0.80, and 11.2% achieve scores between 0.91 and 1.00, indicating many highly efficient producers. However, efficiency scores in the ranges of 0.81–0.90 and 0.91–1.00 are less common, highlighting that while many farmers are pretty efficient, very high efficiency levels are less frequently observed. The distribution reveals that, while most farmers are relatively efficient, there is room for improvement, especially among those at the lower end of the efficiency spectrum. The variation in efficiency underscores the need for targeted interventions and support to help less efficient farmers adopt practices and technologies that can enhance their productivity and bring their performance closer to high-efficiency benchmarks.

### Impact of Adopted Improved Maize Varieties’ (IMVs) on Smallholder Maize Farmers

Table 7 presents the impact of adopting Improved Maize Varieties (IMVs) on smallholder maize farmers, estimated using an endogenous switching regression model. The endogenous switching regression model results reveal a substantial and statistically significant impact of adopting Improved Maize Varieties (IMVs) on smallholder maize farmers regarding maize yields and farm returns. For adopters, the average maize yield is 3.6 metric tonnes/ha, markedly higher than the 1.68 metric tonnes/ha observed for non-adopters, with an Average Treatment Effect (ATE) of 1.92 metric tonnes/ha, supported by a very high t-value of 38.94, indicating a robust and statistically significant improvement. This suggests that IMVs significantly enhance the yields of those who adopt them. Conversely, for non-adopters, the potential yield increases if they had adopted IMVs would be to 3.18 metric tonnes/ha from their current 1.6 metric tonnes/ha, reflecting an ATE of 1.58 metric tonnes/ha and a t-value of 8.34, which, while also statistically significant, indicates a smaller gain compared to current adopters. These results are consistent with literature which indicate that adopting Improved Maize Varieties (IMVs) significantly enhances maize yields and improves the crop’s resilience to changing climatic conditions.^14,67–69^

**Table 7.** Average treatment effects using endogenous switching regression mode.

Outcome variables	Category	Adoption of IMVs	Non-adopters of IMVs	Average Treatment Effect (ATE)	t-value
Maize yields (metric tonnes/ha)	ATT	(a) 3.6 (.37)	(c) 1.68	1.92	38.94**
	ATU	(b) 1.6 (.31)	(d) 3.18	1.58	8.34**
		2.0 (			
Farm Returns (ZAR/ha)	ATT	2574.20	2106.19	468.01***	56.55***
	ATU	2745.43	2288.14	457.29	37.94***

*** and ** are significant at 1% and 5% levels. ATT=Average treatment effect on.

Regarding farm returns, adopters achieve average returns of ZAR 2574.20 per ha, compared to ZAR 2106.19 per ha for non-adopters, with an exceptionally high ATE of ZAR 468.01 per ha and a t-value of 56.55, signifying a substantial and statistically robust increase in returns for those who adopt IMVs. For non-adopters, the potential return increase with IMVs adoption would be to ZAR 2745.43 per ha from their current ZAR 2288.14 per ha, reflecting an ATE of ZAR 457.29 per ha and a t-value of 37.94, confirming that while the increase is smaller than for current adopters, it remains statistically significant. These results highlight that adopting Improved Maize Varieties (IMVs) significantly enhances farm returns and productivity, ultimately leading to substantial improvements in farmers’ livelihoods and overall farm performance.^56,68–71^ These results emphasize that IMVs significantly affect maize yields and farm returns, demonstrating their effectiveness in enhancing agricultural productivity and profitability for current and potential adopters.

### Technical Efficiency and Technology Gap Ratios of Smallholder Farmers

Technical efficiency gains directly lead to higher farm household incomes, with farmers reaping the benefits of these improvements. Therefore, the application of the selectivity-corrected SPF model was necessary. This study utilizes stochastic meta-frontier analysis to assess the technological gaps between the actual outputs observed and the potential outputs each farm could achieve at the highest productive frontier. Consequently, the ensuing discussion is based on the estimation results obtained from the selectivity-corrected SPF model using a matched sample. The study used a meta-frontier for smallholder farmers to conduct a technology gap ratio and technical efficiency. The technology gap ratio quantifies the difference between the performance of a specific maize variety (whether improved or local seeds) and the overall technological advancements accessible to the entire maize industry, considering a range of input factors.^72–74^ The results of TGR are presented in Table 8 below.

**Table 8.** Descriptive statistic of technical efficiency estimates and technology adoption for matched samples.

Technical efficiency and technology gap ratio (TGR)	Mean	Maximum	Minimum	Standard deviation
**Technology gap ratio**				
Improved	0.92	0.99	0.48	0.14
Traditional	0.70	0.99	0.26	0.16
Pooled	0.84	0.99	0.26	0.18
**Technical Efficiency (meta-frontier)**				
Improved	0.76	0.99	0.14	0.22
Traditional	0.42	0.90	0.06	0.20
Pooled	0.54	0.92	0.05	0.21

The study results of technical efficiency estimate and technology adoption for the matched samples provide insightful information on adopting improved versus traditional technologies and their corresponding technical efficiencies. Adopting improved technology (IMVs) is high, with a mean rate of 0.92, though there is some variability, as most farmers opt for improved methods given the challenges farmers face. Traditional technology adoption, with a mean of 0.70, shows more variability, ranging from 0.26 to 0.99, indicating a mix of reliance on traditional and improved methods. On average, farmers adopt a combination of both technologies, as reflected in the pooled mean adoption rate of 0.84, which shows diversity in adoption patterns among smallholder farmers. Despite the widespread use of improved technologies, traditional methods remain common, especially among certain farmers. Overall, there is a clear tendency toward greater adoption of improved technologies, but with notable variability in how farmers choose and implement different farming practices.

Smallholder farmers adopting improved technologies such as maize varieties show a mean technical efficiency of 0.76, with significant variation indicating room for improvement, especially among less efficient farms. In contrast, farms using traditional technologies have a much lower mean efficiency of 0.42, ranging from 0.06 to 0.90, and a high standard deviation of 0.20, reflecting considerable inefficiency. The pooled mean efficiency is 0.54, with values ranging from 0.05 to 0.92, highlighting overall inefficiency across both technology groups. that Farmers which are adopting improved maize varieties are more technically efficient than their counterparts relying on traditional technology or seeds.^72,75–77^ This is attributed to the proper and timely application of recommended inputs such as fertilizers, chemicals, and seeds of improved maize varieties, which are often accompanied by a comprehensive package that includes guidelines on the correct quantities and timing of use, along with other essential cultural practices. The standard deviation of 0.21 underscores the significant disparity in efficiency levels, particularly between farms using improved and traditional technologies. These findings suggest a need for further interventions to improve efficiency, particularly for those relying on traditional methods.

The results in Table 8 highlight a clear distinction between smallholder farmers who adopt improved technologies (IMVs) and those who use traditional practices regarding technology adoption rates and technical efficiency. Smallholder farmers adopting improved technologies show higher mean technical efficiency, indicating that advanced methods contribute to better resource use and productivity. However, significant inefficiencies exist within both groups, especially those using traditional technologies, with notably lower technical efficiency.

## Conclusions

This study empirically investigated the impact of the adoption of Improved Maize Varieties (IMVs) on agricultural productivity and technical efficiency among smallholder farmers in the Eastern Cape, South Africa. The findings highlight the significant role of IMVs in enhancing maize productivity, contributing to improved food security and rural livelihoods. Farmers who adopted IMVs experienced substantially higher yields and farm returns than those who continued using traditional varieties, demonstrating the positive impact of genetic improvements and farmers’ ability to utilize resources better. This superior performance, however, was not uniform across all adopters, underscoring the importance of contextual factors influencing IMV adoption and their effectiveness.

## Relevance of Results and Future Research

The results demonstrate that IMV adoption significantly boosts maize productivity and farm incomes. The robust positive correlation between IMV adoption, enhanced technical efficiency, and positive economic returns underscores their potential to alleviate poverty and food insecurity among smallholder farmers. However, the adoption rates and the effectiveness of IMVs are influenced by numerous factors, including access to complementary inputs, extension services, education, and favorable market conditions. Further investigation is needed to fully quantify how these factors influence the impact of IMVs across varying agroecological contexts and socioeconomic circumstances.

### Future Research Should Explore

Agroecological optimization of IMVs: Investigating optimal IMV varieties for different agroecological zones within the Eastern Cape, considering climate resilience, pest and disease resistance, and soil types.

Longitudinal studies of IMV impact: Tracking the long-term impacts of IMV adoption on farm productivity, household welfare, and sustainability, accounting for external factors like climate change and market fluctuations.

Detailed resource use efficiency: A more in-depth analysis of how different farmer types utilize resources, particularly labor, land, and inputs, to understand variations in technical efficiency and optimize resource allocation strategies. This research could employ more sophisticated econometric methods to address potential endogeneity issues.

Impact of farmer characteristics on adoption: Investigating the influence of other farmer characteristics, such as risk aversion, social capital, and access to information networks, on IMV adoption behavior and outcomes.

Policy impact assessment: Analyzing the effectiveness of various policy interventions, such as input subsidies, credit programs, and agricultural extension services, in promoting IMV adoption and enhancing farm productivity.

## Categorized Recommendations

Based on the study’s objectives, the following recommendations are categorized:

### Practice

Optimal Input Use: Farmers should be trained on effectively using improved maize varieties and associated inputs (fertilizers, pesticides, etc.) to maximize yields and minimize inefficiencies.

Integrated Pest Management: Emphasize integrated pest management strategies to minimize pesticide use while managing pests and diseases effectively.

Improved Weeding Practices: Regular and timely weeding is crucial to optimize resource use and minimize competition from weeds.

### Policy

Strengthen Extension Services: Invest in training and capacity building for agricultural extension officers to provide farmers with adequate support and tailored information.

Improve Access to Inputs: Implement policies to improve the affordability and accessibility of inputs, such as fertilizers, improved seeds, and credit.

Targeted Support: Design targeted programs to assist less efficient farmers with access to resources, knowledge, and credit to enhance productivity.

Climate-Resilient Strategies: Promote climate-smart agricultural practices, such as drought-tolerant maize varieties and improved water management techniques.

### Further Research

Agroecological Matching: Conduct further research to identify the most suitable IMV varieties for specific agroecological zones within the Eastern Cape.

Longitudinal Analysis: Conduct longitudinal studies to assess the long-term impacts of IMV adoption on farm productivity, profitability, and sustainability.

Resource Use Efficiency: Conduct a more detailed analysis of how farmers use resources to pinpoint sources of inefficiency and identify targeted interventions.

Farmer Behavior & Adoption: Investigate the impact of psychological and social factors (risk aversion, social capital, information networks) on adoption decisions and efficiency.

By implementing these recommendations, policymakers, development practitioners, and researchers can work collaboratively to maximize the impact of IMVs on agricultural productivity, food security, and rural livelihoods in the Eastern Cape and other maize-dependent regions.

## References

[1] Food and Agriculture Organization (FAO). Food and agriculture organization of the United Nations. Faostat. 2021;http://faostat.fao.org.

[2] Department of Agriculture, Land Reform and Rural Development [DLRRD]. Economic review of the South African agriculture. 2021. chrome-extension://efaidnbmnnnibpcajpcglclefindmkaj/https://old.dalrrd.gov.za/Portals/0/Statistics%20and%20Economic%20Analysis/Statistical%20Information/Economic%20Review%202021.pdf.

[3] Grain South Africa. Genetic and mechanized innovations in SA maize production. 2024. https://sagrainmag.co.za/2024/11/14/genetic-and-mechanised-innovations-in-sa-maize-production/.

[4] DAgate LA, Lake MW. Seed priming with melatonin improves drought tolerance in maize. J Emerg Invest. 2024;7:1–6. doi: 10.59720/23-097.

[5] Tesfaye K, Gbegbelegbe S, Cairns JE, Shiferaw B, Prasanna BM, Sonder K, Boote K, Makumbi D, Robertson R. Maize systems under climate change in sub-saharan Africa. Int J Clim Change Strategies And Manag. 2015;7(3):247–71. doi: 10.1108/ijccsm-01-2014-0005.

[6] Olalekan AW, Simeon BA. Discontinued use decision of improved maize varieties in Osun state, Nigeria. J Devel And Agric Econ. 2015;7(3):85–91. doi: 10.5897/jdae2014.0573.

[7] Cavane E, Donovan C. Determinants of adoption of improved maize varieties and chemical fertilizers in mozambique. J Int Agric And Ext Educ. 2011;18(3). doi: 10.5191/jiaee.2011.18301.

[8] Collinson S, Hamadziripi E, Ndegwa M, Cairns J, Albertsen M, Ligeyo D, Olsen M, Mashingaidze K, Olsen MS. Incorporating male sterility increases hybrid maize yield in low input African farming systems. Commun Biol. 2022;5(1). doi: 10.1038/s42003-022-03680-7. PMC9307751 35869279

[9] Santpoort R. The drivers of maize area expansion in Sub-Saharan Africa. How policies to boost maize production overlook the interests of smallholder farmers. Land. 2020;9(3):68. doi: 10.3390/land9030068.

[10] Danso-Abbeam G, Bosiako JA, Ehiakpor DS, Mabe FN, Aye G. Adoption of improved maize variety among farm households in the northern region of Ghana. Cogent Econ & Finance. 2017;5(1):1416896–426. 2017.1416896endogenous switching. Proceedings of the American Statistical Association, 5: 423–426. doi:10.1080/23322039.2017.1416896.

[11] Issahaku G, Abdul-Rahaman A. Sustainable land management practices, off-farm work participation and vulnerability among farmers in Ghana: Is there a nexus? Int Soil And Water Conserv Res. 2018;7(1):18–26. doi: 10.1016/j.iswcr.2018.10.002.

[12] Anang BT, Dokyi EO, Asante BO, Donkoh SA. Technical efficiency of resource-poor maize farmers in northern Ghana. Open Agricult. 2022;7(1):69–78. doi: 10.1515/opag-2022-0075.

[13] Anang BT, Alhassan H, Danso-Abbeam G, Yildiz F. Estimating technology adoption and technical efficiency in smallholder maize production: a double bootstrap DEA approach. Cogent Food Agric. 2020;6(1):1833421. doi: 10.1080/23311932.2020.1833421.

[14] Geffersa AG, Agbola FW, Mahmood A. Improved maize adoption and impacts on farm household welfare: evidence from rural Ethiopia. Aus J Agri & Res Econ. 2021;66(4):860–86. doi: 10.1111/1467-8489.12489.

[15] Boadway R, Bruce N. A general proposition on the design of a neutral business tax. J Public Econ. 1984;24(2):231–39. doi: 10.1016/0047-2727(84)90026-4.

[16] Verbeek M. A guide to modern econometrics. Fifth ed. Rotterdam: Rotterdam School of Management, Erasmus University; 2000.

[17] Nyarai Mujuru N, Obi A, Mishi S, Mdoda L. Profit efficiency in family-owned crop farms in Eastern Cape Province of South Africa: a translog profit function approach. Agric & Food Secur. 2022;11(1):2–9. doi: 10.1186/s40066-021-00345-2.

[18] Mdoda L, Obi A, Tamako N, Naidoo D, Baloyi R. Resource use efficiency of potato production among smallholder irrigated farmers in the Eastern Cape Province of South Africa. Sustainability. 2023;15(19):14457. doi: 10.3390/su151914457.

[19] Obi A, Ayodeji BT. Determinants of economic farm-size–efficiency relationship in smallholder maize farms in the Eastern Cape Province of South Africa. Sustainability: Agriculture. 2020;10(4):98. doi: 10.3390/agriculture10040098.

[20] OR Tambo IDP. O.R. Tambo district municipality: integrated development plan (IDP) 2022/27. 2022-2027 [Accessed 2024 Oct 14]. https://lg.treasury.gov.za/supportingdocs/DC15/DC15_IDP%20Final_2023_Y_20220613T155357Z_vuyolwethubisha.pdf.

[21] Alfred Nzo IDP. Alfred Nzo IDP. Alfred Nzo district municipality: final draft integrated development plan: 2022-2027 FY. 2022-2027. 2022-2027 [Accessed 2024 Oct 14]. https://lg.treasury.gov.za/supportingdocs/DC44/DC44_IDP%20Final_2023_Y_20220920T141541Z_bhitshal.pdf.

[22] Aigner DJ, Lovell CAK, Schmidt P. Formulation and estimation of stochastic frontier production function models. J Econom. 1977;6(1):21–37. doi: 10.1016/0304-4076(77)90052-5.

[23] Meeusen W, van den Broeck J. Efficiency estimation from Cobb-Douglas production functions with composed error. Int Econ Rev. 1977;18(2):435–44. doi: 10.2307/2525757.

[24] Ngango J, Hong S. Improving farm productivity through the reduction of managerial and technology gaps among farmers in rwanda. Agric & Food Secur. 2021;10(1):2–14. doi: 10.1186/s40066-020-00284-4.

[25] Farrell MJ. The measurement of productive efficiency. J R Stat Soc Ser A (Gener). 1957;120(3):253–81. doi: 10.2307/2343100.

[26] Fraser I, Cordina D. An application of data envelopment analysis to irrigated dairy farms in Northern Victoria, Australia. Agric Syst. 1999;59(3):267–82. doi: 10.1016/S0308-521X(99)00009-8.

[27] Mango N, Mapemba L, Tchale H, Makate C, Dunjana N, Lundy M, Elliott C. Comparative analysis of tomato value chain competitiveness in selected areas of Malawi and Mozambique. Cogent Econ & Finance. 2015;3(1):1088429. doi: 10.1080/23322039.2015.1088429.

[28] Belete AA. Determinants of market participation of smallholder sorghum farmers and strategies for improving their participation: the case of Moretna Jiru District, Ethiopia. Research Square. 2020;1–26.

[29] Ali I, Xue-Xi H, Khan I, Ali H, Baz K, Khan SU. Technical efficiency of hybrid maize growers: a stochastic frontier model approach. J Intgr Agricult. 2019;18(10):2408–21.

[30] Battese GE, Corra GS. Estimation of a production frontier model: with application to the pastoral zone of eastern Australia. Australian J Agric Econ. 1977;21(3):169–79. doi: 10.1111/j.1467-8489.1977.tb00204.x.

[31] Adego T, Simane B, Woldie A. The impact of adaptation practices on crop productivity in northwest Ethiopia: an endogenous switching estimation. Devel Stud Res. 2019;6(1):129–41. doi: 10.1080/21665095.2019.1678186.

[32] Ogunleye A, Kehinde A, Mishra A, Ogundeji A. Impacts of farmers’ participation in social capital networks on climate change adaptation strategies adoption in Nigeria. Heliyon. 2021;7:e08624. doi: 10.1016/j.heliyon.2021.e08624. 35005276 PMC8715181

[33] Di Falco S, Veronesi M. Managing environmental risk in the presence of climate change: the role of adaptation in the nile basin of Ethiopia. In: Lipper L, McCarthy N, Zilberman D, Asfaw S Branca G, editors. Climate smart agriculture. Natural resource management and policy. Vol. 52. Cham: Springer; 2018. p. 497–526.

[34] Khanal U, Wilson C, Hoang V, Boon Lee B. Farmers’ adaptation to climate change, its determinants and impacts on rice yield in Nepal. Ecol Econ. 2018;144:139–47. doi: 10.1016/j.ecolecon.2017.08.006.

[35] Atube F, Malinga GM, Nyeko M, Okello DM, Alarakol SP, Okello-Uma I. Determinants of smallholder farmers’ adaptation strategies to the effects of climate change: evidence from northern Uganda. Agric & Food Secur. 2021;10(1):2–14. doi: 10.1186/s40066-020-00279-1.

[36] Mdoda L, Obi A, Ncoyini-Manciya Z, Christian M, Mayekiso A. Assessment of profit efficiency for spinach production under small-scale irrigated agriculture in the Eastern Cape Province, South Africa. Sustainability. 2022;14(5):2991. doi: 10.3390/su14052991.

[37] Abegunde VO, Sibanda M, Obi A. Effect of climate-smart agriculture on household food security in small-scale production systems: a micro-level analysis from South Africa. Cogent Soc Sci. 2022;8(1):2086343. doi: 10.1080/23311886.2022.2086343.

[38] Aswathy N, Joseph I. A logit analysis of the factors affecting cage fish farming adoption decisions in the Southwest Coast of India. Curr J Appl Sci Technol. 2020;39(40):29–34. doi: 10.9734/CJAST/2020/v39i4031109.

[39] Sigigaba M, Mdoda L, Mditshwa A. Adoption drivers of improved open-pollinated (OPVs) maize varieties by smallholder farmers in the Eastern Cape Province of South Africa. Sustainability. 2021;13(24):13644. doi: 10.3390/su132413644.

[40] Mdoda L, Mdletshe STC, Dyiki MC, Gidi LS. The impact of agricultural mechanization on smallholder agricultural productivity: evidence from mnquma local municipality in the Eastern Cape Province. S Afr Jnl Agric Ext. 2022;50(1):76–101s. doi: 10.17159/2413-3221/2022/v50n1a11218.

[41] Shahzad MF, Abdulai A. Adaptation to extreme weather conditions and farm performance in rural Pakistan. Agric Syst. 2020;180:102772. doi: 10.1016/j.agsy.2019.102772.

[42] Lokshin M, Sajaia Z. Maximum likelihood estimation of endogenous switching regression models. Stata J. 2004;4(3):282–89. doi: 10.1177/1536867X0400400306.

[43] Tanimonure VA, Naziri D, Codjoe SNA, Ayanwale AB. Underutilised indigenous vegetables for household dietary diversity in Southwest Nigeria. Agriculture. 2021;11(11):1064. doi: 10.3390/agriculture11111064.

[44] Abdulai A, Huffman W. The adoption and impact of soil and water conservation technology: an endogenous switching regression application. Land Econ. 2014;90(1):26–43. doi: 10.3368/le.90.1.26.

[45] Maddala G. Limited-dependent and qualitative variables in econometrics. Cambridge: Cambridge University Press; 1983. doi: 10.1017/CBO9780511810176.

[46] Heckman J. Sample selection bias as a specification error. Econometrica. 1979;47(1):153–61. doi: 10.2307/1912352.

[47] Olasehinde TS, Qiao F, Mao S. Impact of improved maize varieties on production efficiency in Nigeria: separating technology from managerial gaps. Sustainability: Agriculture. 2023;13(3):611. doi: 10.3390/agriculture13030611.

[48] Mdoda L, Meleni S, Mujuru N, Alaka KO. Agricultural credit effects on smallholder crop farmers input utilisation in the Eastern Cape Province, South Africa. J Hum Ecol. 2019;66(1–3):45–55.

[49] Makamane A, Van Niekerk J, Loki O, Mdoda L. Determinants of climate smart-agriculture (CSA) technologies adoption by smallholder food crop farmers in mangaung metropolitan municipality, free state. S Afr Jnl Agric Ext. 2023;51(4):52–74. doi: 10.17159/2413-3221/2023/v51n4a1645.

[50] Mukarumbwa P, Taruvinga A. Landrace and GM maize cultivars’ selection choices among rural farming households in the Eastern Cape Province, South Africa. GM Crops & Food. 2023;14(1):1–15. doi: 10.1080/21645698.2023.2215146. PMC10202092 37210729

[51] Mdoda L, Christian M, Agbugba I. Use of information systems (Mobile phone app) for enhancing smallholder farmers’ productivity in Eastern Cape Province, South Africa: implications on food security. J Knowl Econ. 2024;15(1):1993–2009. doi: 10.1007/s13132-023-01212-0.

[52] Oluwayemisi I, Olarinde L, Fatunbi A. Determinants of adoption of improved maize varieties in Kano-Katsina-Maradi, west Africa. Afr Crop Sci J. 2017;25(1):1. doi: 10.4314/acsj.v25i1.1s.

[53] Dokyi E, Anang BT, Owusu V. Impacts of improved seed maize technology adoption on productivity and technical efficiency in Northern Ghana. Open Econ. 2021;4(1):118–32. doi: 10.1515/openec-2020-0102.

[54] Ngcinela S. Smallholder farmers’ selection criteria of maize varieties in Eastern Cape Province (implications for adoption of GM maize): the case of port St. Johns, Flagstaff, and Mqanduli [Published MSc dissertation]. Alice, South Africa: University of Fort Hare; 2018.

[55] Chimonyo) Mutengwa CS, Chiduza C, Tandzi LN, Chimonyo VGP. Participatory variety selection of maize genotypes in the Eastern Cape Province of South Africa. S Afr Jnl Agric Ext. 2019;47(1):103–17. doi: 10.17159/2413-3221/2019/v47n1a493.

[56] Merga G, Sileshi M, Zeleke F. Welfare impact of improved maize varieties adoption among smallholder farmers in Amuru District of Horo Guduru Wollega, Ethiopia. Cogent Econ And Finance. 2023;11(1):2207923. doi: 10.1080/23322039.2023.2207923.

[57] Onuwa GC, Mailumo SS, Oyewole SO. Socio-economic determinants of adoption of maize production technologies among smallholders. Agriekonomika Jurnal Sosial EKonomi dan Pertanian. 2023;12(1):83–94. doi: 10.21107/agriekonomika.v12i1.14621.

[58] Keneni K, Beyene F, Haji J, Lemma T. Adoption and impact of improved maize varieties on smallholder farmers’ farm productivity and net-income in Eastern Ethiopia. Int J Novel Res And Devel. 2018;7(10):a905–20.

[59] Ndeko AB, Chuma GB, Mondo JM, Kazamwali LM, Mugumaarhahama Y, Bisimwa EB, Mushagalusa GN. Farmers’ preferred traits, production constraints, and adoption factors of improved maize varieties under South-Kivu rainfed agro-ecologies, Eastern D.R. Congo: Implication for maize breeding. Research Square; 2022. p. 3–41. doi: 10.21203/rs.3.rs-1893945/v1.

[60] Sánchez-Toledano BI, Kallas Z, Rojas OP, Gil JM. Determinant factors of the adoption of improved maize seeds in Southern Mexico: a survival analysis approach. Sustainability. 2018;10(10):3543. doi: 10.3390/su10103543.

[61] Wossen T, Abdoulaye T, Alene A, Feleke S, Menkir A, Manyong V. Measuring the impacts of adaptation strategies to drought stress: the case of drought tolerant maize varieties. J Environ Manag. 2017;203:106–13. doi: 10.1016/j.jenvman.2017.06.058. PMC5607453 28779600

[62] Rahman KMM, Mia MI, Alam MA. Farm-size-specific technical efficiency: a stochastic frontier analysis for rice growers in Bangladesh. Bangladesh J Agric Econ. 2012;XXXV(1&2):131–42.

[63] Abdulai S, Nkegbe PK, Donkoh SA. Assessing the technical efficiency of maize production in northern Ghana: the data envelopment analysis approach. Cogent Food Agric. 2018;4(1):1512390.

[64] Phali L, Mudhara M, Ferrer S, Makombe G. Evaluation of water-user performance in smallholder irrigation schemes in KwaZulu-Natal Province, South Africa: a stochastic meta-frontier analysis. Front Sustain Food Syst. 2022;6:1022410. doi: 10.3389/fsufs.2022.1022410.

[65] Ferreira T. Does Education Enhance Productivity in Smallholder farming in Johanessburg. Stellenbosch Economic Working Papers: WP05/2018. 2018. www.ekon.sun.ac.za/wpapers/2018/wp052018.

[66] Danso-Abbeam G, Ehiakpor DS, Aidoo R. Agricultural extension and its effects on farm productivity and income: insight from Northern Ghana. Agric & Food Secur. 2018;7(1):1–10.

[67] Amondo E, Simtowe F, Rahut DB, Erensteinand O. Productivity and production risk effects of adopting drought-tolerant maize varieties in Zambia. Int J Clim Change Strategies And Manag. 2019;11(4):570–91. doi: 10.1108/IJCCSM-03-2018-0024. PMC7774807 33408756

[68] Keneni K, Beyene F, Haji J, Lemma T. Adoption and impact of improved maize varieties on smallholder farmers’ farm productivity and net-income in Eastern Ethiopia. Int J Novel Res And Devel. 2022;7(10):a905–20.

[69] Abdoulaye T, Wossen T, Awotide B. Impacts of improved maize varieties in Nigeria: ex-post assessment of productivity and welfare outcomes. Food Sec. 2018;10(2):369–79. doi: 10.1007/s12571-018-0772-9.

[70] Jaleta M, Kassie M, Marenya P, Yirga C, Erenstein O. Impact of improved maize adoption on household food security of maize producing smallholder farmers in Ethiopia. Food Sec. 2018;10(1):81–93. doi: 10.1007/s12571-017-0759-y.

[71] Gebre GG, Mawia H, Makumbi D, Rahut DB. The impact of adopting stress-tolerant maize on maize yield, maize income, and food security in Tanzania. Food And Energy Secur. 2021;10(4):313. doi: 10.1002/fes3.313. PMC9285390 35860337

[72] Muzekenyi M, Zuwarimwe J, Kilonzo BM. Analysis of technical efficiency of small-scale commercial farmers in Vhembe district. S Afr Jnl Agric Ext. 2021;49(1):91–104. doi: 10.17159/2413-3221/2021/v49n1a10780.

[73] Gebre G, Isoda H, Rahut D, Amekawa Y, Nomura H. Gender differences in the adoption of agricultural technology: the case of improved maize varieties in southern Ethiopia. Women S Stud Int Forum. 2019;76:102264. doi: 10.1016/j.wsif.2019.102264. PMC6894305 31853161

[74] Alem H, Lien G, Hardaker JB, Guttormsen A. Regional differences in technical efficiency and technological gap of Norwegian dairy farms: a stochastic meta-frontier model. Appl Econ. 2018;51(4):1–14. doi: 10.1080/00036846.2018.1502867.

[75] Matthew N, Conradie B, Piesse J. Technological differences in South African sheep production: a stochastic meta-frontier analysis. Agrekon. 2023;62(1):19–30. doi: 10.1080/03031853.2022.2149577.

[76] Kodua TT, Onumah EE, Mensah-Bonsu A. Technical efficiency of improved and local variety seed maize farms in Ghana: a meta-frontier analysis. Cogent Econ & Finance. 2022;10(1):2022858. doi: 10.1080/23322039.2021.2022858.

[77] Anang BT, Bäckman S, Rezitis A. Production technology and technical efficiency: irrigated and rain-fed rice farms in northern Ghana. Eurasian Econ Rev. 2017;7(1):95–113. doi: 10.1007/s40822-016-0060-y.

