# Correction

**DOI:** 10.1080/21645698.2025.2526997

**Published:** 2025-07-11

**Article title**: Evaluating the impact of improved maize varieties on agricultural productivity and technical efficiency among smallholder farmers in the Eastern Cape, South Africa: an empirical analysis

**Authors:**

Lelethu Mdoda, Nthabeleng Tamako, Lungile S. Gidi, & Denver Naidoo

**Journal**: *Biotechnology in Agriculture and the Food Chain*

**Bibliometrics**: Volume 16, Number 01, pages 272 - 304

**DOI**: http://dx.doi.org/ 10.1080/21645698.2025.2476667

Kg/ha should have been metric tonnes/ha in the original article.

This has been corrected in the online publication.

